# Non-apoptotic function of caspase-8 confers prostate cancer enzalutamide resistance via NF-κB activation

**DOI:** 10.1038/s41419-021-04126-4

**Published:** 2021-09-04

**Authors:** Jia Xia, Jiahui Zhang, Liangzhe Wang, Hailong Liu, Jie Wang, Junyan Liu, Zhaoqian Liu, Yingjian Zhu, Yingjie Xu, Wen Yang, Yongjiang Yu

**Affiliations:** 1 Department of Urology, Xinhua Hospital, Shanghai Jiao Tong University School of Medicine, Shanghai, P.R. China; 2 Department of Nephrology, Renji Hospital, School of Medicine, Shanghai Jiao Tong University, Shanghai, P.R. China; 3 Department of Biochemistry and Molecular & Cell Biology, Shanghai Jiao Tong University School of Medicine, Shanghai, P.R. China; 4 Department of Pathology, Changzheng Hospital, Naval Medical University, Shanghai, P.R. China; 5 Department of Orthopaedics, Xiangya Hospital, Central South University, Changsha, P.R. China; 6 Department of Clinical Pharmacology, Xiangya Hospital, Central South University, Changsha, P.R. China; 7 State Key Laboratory of Oncogenes and Related Genes, Shanghai Jiao Tong University School of Medicine, Shanghai, P.R. China

**Keywords:** Prostate cancer, Apoptosis

## Abstract

Caspase-8 is a unique member of caspases with a dual role in cell death and survival. Caspase-8 expression is often lost in some tumors, but increased in others, indicating a potential pro-survival function in cancer. By analyzing transcriptome of enzalutamide-resistant prostate cancer cells, we found that resistance was conferred by a mild caspase-8 upregulation that in turn led to NF-κB activation and the subsequent upregulation of the downstream IL-8. Mechanistically, we found that the pro-survival and enzalutamide-resistance-promoting features of caspase-8 were independent of its proteolytic activity, using a catalytically-inactive caspase-8 mutant. We further demonstrated that caspase-8 pro-apoptotic function was inhibited via cFLIP binding. Moreover, high caspase-8 expression was correlated with a worse prognosis in prostate cancer patients. Collectively, our work demonstrates that enzalutamide-resistance is mediated by caspase-8 upregulation and the consequent increase in NF-κB/IL-8 mediated survival signaling, highlighting caspase-8 and NF-κB as potential therapeutic targets to overcome enzalutamide-resistance in CRPC.

## Introduction

Prostate cancer (PCa) is the fifth leading cause of cancer-related death and the second most frequently diagnosed malignancy in men [1]. Enzalutamide, a second-generation AR antagonist, has been widely used for the treatment of metastatic castration-resistant prostate cancer (CRPC), prolonging CRPC patients survival [2, 3]. However, about 25% of CRPC patients are primarily resistant to enzalutamide, while others, initially sensitive, develop enzalutamide resistance (ENZR) [4]. This notion, together with the expanding enzalutamide therapeutic indications [5], makes a complete understanding of ENZR mechanisms an urgent matter. Several ENZR mechanisms have been reported in PCa. Most of them are androgen-dependent, including increased intracrine androgen effects and AR aberrant activation due to AR mutations or splicing variants [6, 7]. However, nonandrogen-dependent pathways also play a pivotal role in ENZR [8].

Caspases are cysteine proteases that initiate various programmed cell death pathways. Caspase-8 is a peculiar member of this family: an initiator caspase, implicated in both the extrinsic apoptosis and necroptosis suppression [9, 10]. In the regulation of tumor cell survival, caspase-8 also plays a controversial role. Both the absence and expression of caspase-8 were involved in tumor invasion and metastasis in cancers [11, 12]. For instance, in some cancers, including hepatocellular carcinoma, pancreatic carcinoma, caspase-8 was upregulated, compared to normal tissues [13]. Interestingly, some studies demonstrated that the role of caspase-8 in cancer is independent of its proteolytic activity [12, 14]. In certain cancer cells, it has been suggested that caspase-8 sustains tumor survival by regulating the “FADDosome” complex assembly and promoting proinflammatory cytokines secretion [15]. However, little is known about the role of caspase-8 in the context of antitumor therapy.

The canonical NF-κB (p65/p50) pathway is essential for the regulation of cytokine production [16]. Importantly, this pathway is often constitutively activated in PCa. It plays a critical role in tumor growth and metastasis and contributes to cancer progression to the late androgen-independent stage [17]. However, the role of NF-κB signaling in enzalutamide-resistant PCa remains to be illustrated.

To address the abovementioned knowledge gaps, we analyzed the transcriptome of enzalutamide-resistant PCa cell lines [18]. Remarkably, we found that compared to enzalutamide-sensitive PCa cells, caspase-8 was upregulated in ENZR ones. We identified an unanticipated caspase-8 role in promoting a continuous activation of NF-κB pathway (instead of inducing apoptosis). This NF-κB activation led to increased IL-8 levels, which a recognized pro-survival function. To our knowledge, this is the first study to highlight the involvement of caspase-8 cell death-independent effects in the promotion of ENZR.

## Results

### Systematic identification of ENZR mechanisms in PCa by transcriptomic analysis

**Fig. 1:**
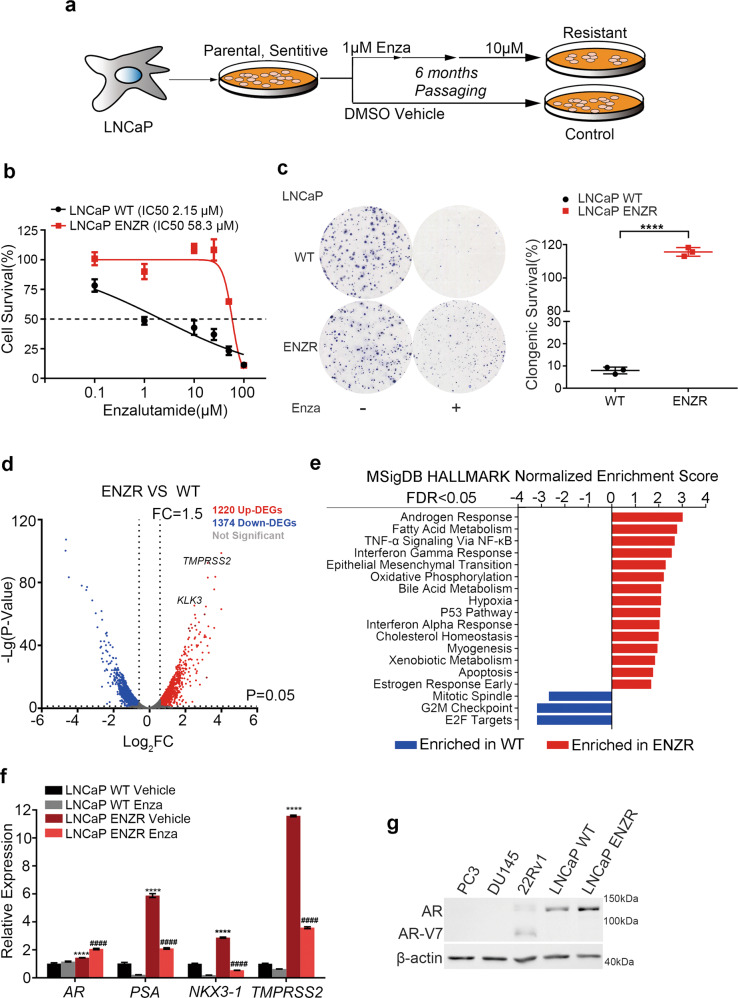
Enzalutamide-resistance mechanisms identified by transcriptomic analysis in prostate cancer cells. **a** Schematic diagram representing the establishment of LNCaP ENZR cell lines. LNCaP parental cells were treated with enzalutamide (1 μM to 10 μM) for 6 months. LNCaP parental cells were cultured in parallel**. b** Survival rate curves for LNCaP ENZR and WT treated with enzalutamide. Briefly, 1 × 10^3^ cells were seeded in 96-well plates and after 24 h, adherent cells were treated with growth medium supplemented with indicated concentrations of enzalutamide. After 7-day treatment, cell viability was analyzed using CCK-8 and normalized to the drug-free condition. The IC_50_ of LNCaP WT and ENZR was 2.15 and 58.3 μM, respectively (*n* = 3). **c** LNCaP WT and ENZR Cells were seeded in 6-well plates with 2 × 10^3^ cells per well and treated with or without 1 μM enzalutamide for 14 days. Then, cells were fixed with 4% PFA, stained with 0.1% crystal violet and the numbers of colonies were counted using Image J. Clongenic survival rate were normalized to drug-free control (*n* = 3). **d** Volcano plots illustrating DEGs in LNCaP ENZR compared to WT cells. DEGs were defined based on the cutoff threshold of *P* value < 0.05 and FC > 1.5. Upregulated DEGs are highlighted in red, while downregulated DEGs are represented in blue. The *x*- and *y*-axes represent average Log_2_(FC) and the -Lg(*P* value), respectively. Dotted lines indicate the 1.5-fold change and 0.05-*P* value cutoffs. In total, 1220 upregulated and 1374 downregulated genes were screened, *KLK3(PSA)*, *TMPRSS2* were highlighted. **e** Gene set enrichment analysis (GSEA) of LNCaP ENZR and WT cells. Enriched pathways in LNCaP WT and ENZR were defined using the GSEA software: DEGs data from RNA-Seq output were calculated for enriched pathways using hallmarks gene set collection. Pathways enriched in LNCaP ENZR (right) or in LNCaP WT cells (left) were ranked by normalized enrichment score (NES), FDR < 0.05. Androgen Response stood out as the top one enriched pathway in ENZR. **f** Relative expression of *AR* and *AR* target genes (*PSA*, *TMPRSS2*, and *NKX3-1*) were analyzed by RT-qPCR under the condition of LNCaP ENZR and WT cells treated with or without 10 μM enzalutamide for 48 h. *GAPDH* was used as internal reference. *vs LNCaP WT vehicle, #*vs* LNCaP WT Enza. *n* = 9 from three independent experiments. **g** Western blot analysis protein level of AR and AR-V7 in prostate cell lines (PC3, DU145, 22Rv1, LNCaP WT, and LNCaP ENZR). Data are represented as Mean ± SEM. Statistical relevance was assessed using the Student’s *t* test and is represented: *P < 0.05; **P < 0.01; ***P < 0.001; ****P < 0.0001.

To establish an acquired enzalutamide-resistant cell model, LNCaP cells were treated with enzalutamide progressively increasing doses in a 6-month period (Fig. 1a). Cell morphology changed, and LNCaP ENZR cells became elongated and disarranged (Fig. S1a). The half-maximal inhibitory concentration (IC_50_) determined for enzalutamide was 27-fold higher in LNCaP ENZR, compared to LNCaP WT (58.3 μM *vs* 2.15 μM) (Fig. 1b). Colony formation assay further indicated a greater LNCaP ENZR proliferation ability than LNCaP WT under enza-treatment, normalized to DMSO-treated cells (Fig. 1c). Cell proliferation assay showed reduced sensitivity to enzalutamide in LNCaP ENZR and 22Rv1 (Fig. S1b–d).

To identify the pathways and possible molecular resistance determinants in ENZR, we performed a comparative transcriptomic analysis of LNCaP ENZR and WT cells. Differentially expressed genes (DEGs) were defined as ones with an expression fold change (FC) > 1.5 (and a *P* < 0.05). 1220 upregulated and 1374 downregulated genes were identified in LNCaP ENZR cells, as represented in the volcano plot (Fig. 1d and Dataset S1). Gene Set Enrichment Analysis (GSEA) revealed several pathways significantly enriched in LNCaP ENZR samples. Among them, Androgen Response, Fatty Acid Metabolism, TNF-α signaling via NF-κB, Interferon-γ Response, and Epithelial-Mesenchymal Transition (EMT) pathways ranked as the top five (FDR < 0.05) (Fig. 1e and Fig. S1e). Importantly, Androgen Response and EMT have been previously reported as mechanisms for ENZR [19–21]. Remarkably, in our study, the mRNA levels of full-length AR (AR-FL) and downstream genes including *KLK3(PSA)*, *TMPRSS2*, *NKX3-1* were significantly upregulated, indicating the higher AR activity in LNCaP ENZR compared to LNCaP WT (Fig. 1f). Western blot further confirmed higher AR expression in LNCaP ENZR compared to LNCaP WT (Fig. 1g). Importantly, in another enzalutamide-resistant PCa cell line VCaP (VCaP ENZR, Fig. S1f), the protein and mRNA level of both AR-FL and its splice variant V7 (AR-V7) were significantly increased, together with upregulation of *PSA, TMPRSS2* (Fig. S1g–i). Taken together, these results attest to the successful establishment of ENZR in vitro model and highlight the reliability of our RNA-seq data.

### Upregulated caspase-8 promotes enzalutamide-resistance in LNCaP cells

**Fig. 2:**
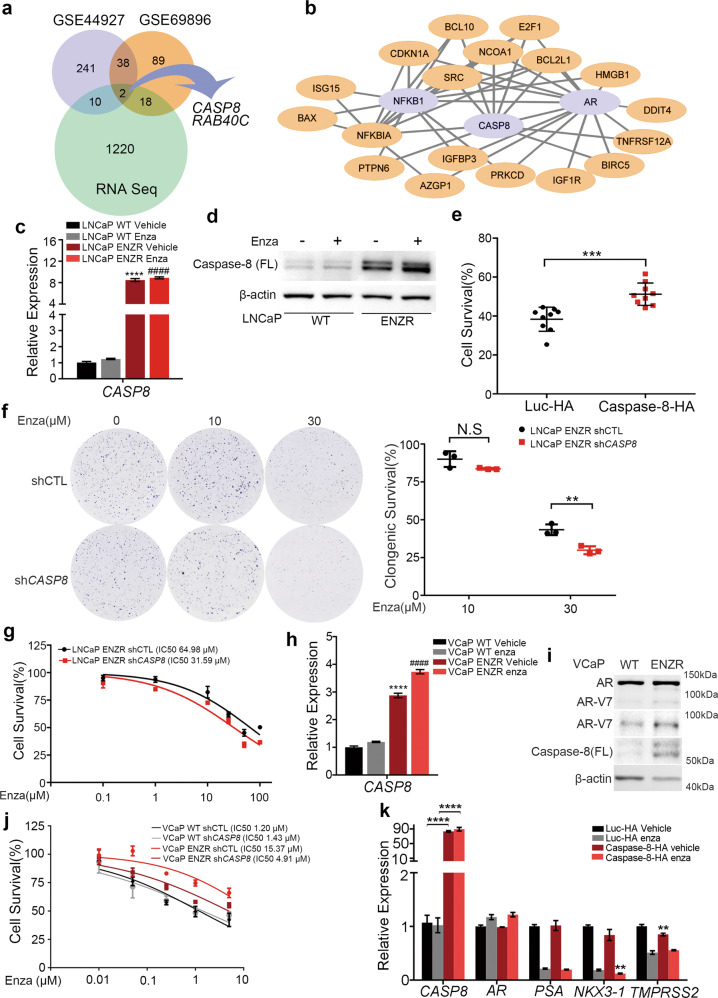
Integrated transcriptomic analysis reveals caspase-8 as a pro-enzalutamide-resistance factor. **a** Venn diagram representing the integrated transcriptomic analysis. Upregulated genes in the context of enzalutamide-resistant cell lines from three datasets were analyzed (our RNA seq results, and GSE44927 and GSE69896 dataset). Only two genes (*RAB40C* and *CASP8*) were commonly upregulated in three databases. **b** AR/NF-κB/caspase-8 interaction networks. Differentially expressed genes’ interactions were evaluated using Ingenuity Pathway Analysis. Twenty caspase-8 related genes have known relationships with NF-κB or AR pathways. The network was drawn by Cytoscape. **c** RT-qPCR analysis of *CASP8* relative expression in LNCaP ENZR and WT cells treated with or without 10 μM enzalutamide for 48 h. *GAPDH* was used as internal reference. *vs LNCaP WT vehicle, #*vs* LNCaP WT Enza. *n* = 9 from three independent experiments. **d** Western blot analysis protein level of Caspase-8 in LNCaP ENZR and WT cells treated with or without 10 μM enzalutamide for 48 h. **e** Cell survival (%) of LNCaP overexpressing caspase-8-HA under enzalutamide treatment. 5 × 10^4^ LNCaP caspase-8-HA and luc-HA cells were seeded in 6-well plates and treated with 10 μM enzalutamide or DMSO next day for 8 days with 2% FBS in culture medium. After 8-day-treatment, cells were harvested and counted. Surviving cells were normalized to DMSO-treated control condition. LNCaP luc-HA was used as a control. n = 9 from three independent experiments. **f** LNCaP ENZR shCTL and sh*CASP8* cells were seeded in 6-well plates with 2 × 10^3^ cells per well and treated with or without 10/30 μM enzalutamide for 14 days. Then, cells were fixed with 4% PFA, stained with 0.1% crystal violet, and the numbers of clonies were counted using Image J. Clongenic survival rate were normalized to drug-free control (*n* = 3). **g** Survival rate curves of LNCaP ENZR sh*CASP8* and LNCaP ENZR shCTL cells treated with enzalutamide. Briefly, 3 × 10^3^ cells were seeded in 96-well plates and after 24 h, adherent cells were treated with a growth medium supplemented with indicated concentrations of enzalutamide. After 7-day treatment, cell viability was analyzed using CCK-8 and normalized to the drug-free condition. The IC_50_ of LNCaP ENZR shCTL and sh*CASP8* was 64.98 and 31.59 μM, respectively (*n* = 3). **h** RT-qPCR analysis of relative expression of *CASP8* under the condition of VCaP ENZR and WT cells treated with or without 1 μM enzalutamide for 48 h. *GAPDH* was used as internal reference. *vs VCaP WT vehicle, #*vs* VCaP WT Enza (*n* = 3). **i** Western blot analysis protein level of Caspase-8 and AR in VCaP ENZR and WT cell line. **j** Survival rate curves for caspase-8 knocking down VCaP ENZR and VCaP CTL cells. Briefly, 2 × 10^4^ cells of VCaP ENZR shCTL, ENZR sh*CASP8*, CTL shCTL, and CTL sh*CASP8* were seeded in 96-well plates and left to adhere for 24 h. Then, cells were treated with indicated concentrations of enzalutamide for 2 days. Cell viability was analyzed using CCK-8 and normalized to the drug-free condition. The IC_50_ of VCaP ENZR shCTL, ENZR sh*CASP8*, CTL shCTL, and CTL sh*CASP8* was 15.37, 4.91, 1.20, and 1.43 μM, respectively (*n* = 3). **k** RT-qPCR analysis of *AR* and *AR* target genes relative expression in LNCaP caspase-8-HA. Cells were treated with 10 μM enzalutamide or DMSO vehicle for 48 h. *AR* and *AR*-targeted genes (*PSA*, *NKX3-1*, *TMPRSS2*) were compared between LNCaP luc-HA and caspase-8-HA cells. *GAPDH* was used as internal reference. *n* = 9 from three independent experiments.

Due to the heterogeneity of PCa cells, we sought to identify candidate genes potentially involved in ENZR across various genetic backgrounds to improve the reliability. We selected another two ENZR-related transcriptomics datasets from Gene Expression Omnibus (GEO) (GSE69896 and GSE44927) [22, 23]. Considering the three datasets, *CASP8* and *RAB40C* mRNAs were upregulated in all of them (Fig. 2a). Furthermore, an Ingenuity Pathway Analysis (IPA) was performed to explore co-upregulated genes. Interestingly, *CASP8* displayed a strong affinity with 20 other known genes that simultaneously interacted with AR or NF-κB signaling pathways (enriched in our LNCaP ENZR cells, as abovementioned) (Fig. 2b). In contrast, *RAB40C* was not associated with any known genes important for top hits of GSEA (Data not shown). The mRNA and protein level of caspase-8 were significantly upregulated in LNCaP ENZR, compared to LNCaP WT (*P* < 0.0001 for mRNA level) (Fig. 2c–d). These results confirm the correlation between high levels of caspase-8 and ENZR.

To prove the involvement of caspase-8 in ENZR, caspase-8 with a C-terminal HA tag (caspase-8-HA) was overexpressed in LNCaP WT, while caspase-8 was stably knocked down in LNCaP ENZR using shRNA (Fig. S2a, b). We found that the transient excessive accumulation of caspase-8 in LNCaP WT resulted in a dramatic induction of apoptosis (Fig. S2c, d), while the moderate (but stable) caspase-8 upregulation resulted in lower apoptosis levels. Remarkably, the overexpression of caspase-8-HA improved the WT cells’ viability under enzalutamide treatment, compared to LNCaP WT overexpressing luciferase-HA (LNCaP luc-HA) (52 *vs* 38%, normalized to DMSO-treated cells; *P* < 0.001) (Fig. 2e). Consistently, knockdown of caspase-8 in LNCaP ENZR increased susceptibility to enzalutamide treatment as determined by colongenic survival assay (Fig. 2f) and CCK-8 assay: IC_50_ of 64.98 μM *vs* 31.59 μM for LNCaP ENZR shCTL and sh*CASP8*, respectively (Fig. 2g). Meanwhile, downregulation of caspase-8 in LNCaP luc-HA also led to lower survival rates, further suggesting the pro-survival role of caspase-8 under enzalutamide treatment (Fig. S2e, f). Consistently, higher mRNA and protein level of caspase-8 were found in VCaP ENZR cells compared to WT (Fig. 2h, i); while knockdown of caspase-8 sensitized VCaP ENZR cells to enzalutamide (IC_50_ of 15.37 μM *vs* 4.91 μM for VCaP ENZR shCTL and sh*CASP8*, Fig. 2j and Fig. S2g, h). These results further indicate the role of caspase-8 in conferring ENZR.

Since enzalutamide inhibits the AR pathway, we investigated if caspase-8-induced ENZR was due to an aberrant AR pathway activation. We found that upon caspase-8 overexpression in LNCaP WT, no significant upregulations (with FC > 1.5) were observed with respect to AR and its downstream genes expression, both in the presence or absence of enzalutamide (Fig. 2k). Taken together, our results suggest that caspase-8 mediates ENZR via AR-independent mechanisms.

### Caspase-8 promotes enzalutamide resistance via NF-κB activation

**Fig. 3:**
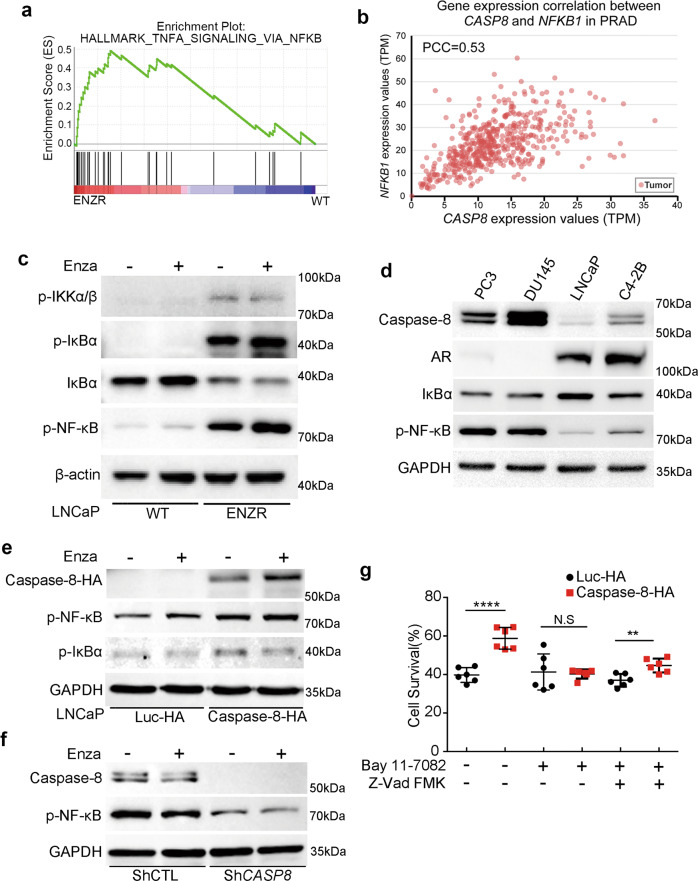
Caspase-8 promotes enzalutamide resistance through NF-κB activation. **a** Gene Set Enrichment Analysis of DEGs profile in LNCaP WT and ENZR cells against TNF-α signaling via NF-κB. The normalized enrichment score was 2.682 and FDR q-value < 1 × 10^–5^. **b** Correlation of *CASP8* and *NFKB1* gene expression in PRAD. The TPM of *CASP8* and *NFKB1* showed an intermediate correlation (PCC = 0.53). **c** Western blot analysis phosphorylation level of IKKα/β, IκBα, and NF-κB, and protein level of IκBα in LNCaP ENZR cells. Cells were treated with 10 μM enzalutamide or DMSO vehicle for 24 h. **d** Western blot analysis protein level of caspase-8, AR, IκBα and phosphorylation level of NF-κB in PC3, DU145, LNCaP, and C4-2B prostate cancer cells. **e** Western blot analysis protein level of caspase-8, and phosphorylation level of NF-κB and IκBα under caspase-8 overexpression. LNCaP luc-HA and caspase-8-HA cells were treated with 10 μM enzalutamide or DMSO vehicle and serum-starved for 24 h. **f** Western blot analysis protein level of caspase-8, and phosphorylation level of NF-κB under caspase-8 silencing. LNCaP ENZR ShCTL and Sh*CASP8* cells were treated with 10 μM enzalutamide or DMSO vehicle and serum-starved for 24 h. **g** Cell viability (%) of LNCaP caspase-8-HA cells were analyzed under enzalutamide treatment. 5 × 10^4^ of LNCaP caspase-8-HA and LNCaP luc-HA cells were seeded in 6-well plates. After 24 h, adherent cells were treated with 10 μM enzalutamide or DMSO vehicle under the condition of 1 μM Bay 11–7082 (inhibitor of IκBα) and 20 μM Z-Vad FMK (multi-inhibitor of caspase) for 8 days with 2% FBS in culture medium, cells were harvested and counted. Surviving cells were normalized to DMSO-treated control condition. LNCaP luc-HA was used as a control. *n* = 6 from three independent experiments.

Based on the abovementioned GSEA, we investigated the relation between caspase-8 and the NF-κB pathway in PCa cell lines (Figs. 2b and 3a, b). Consistently, we detected higher levels of IKKα/ß, IκBα and NF-κB(p65) phosphorylation and lower levels of IκBα in LNCaP ENZR, compared to LNCaP WT (Fig. 3c), indicating the NF-κB pathway activated in the context of ENZR. In line with this finding, higher level of NF-κB(p65) phosphorylation was observed in VCaP ENZR, compared to VCaP WT (Fig. S3a). Interestingly, we found that also in other AR-negative cell lines (PC3 and DU145) resistant to enzalutamide treatment, NF-κB phosphorylation was increased and IκBα levels were decreased (Fig. 3d). In contrast, androgen-dependent cell lines, such as LNCaP WT and C4-2B generally displayed lower levels of basal NF-κB activation (Fig. 3d). These observations suggest that NF-κB activation is an alternative pathway supporting cell growth (independently of AR-signaling) in PCa cells.

In line with the NF-κB activation statuses mentioned above, the expression levels of caspase-8 were also higher in AR-negative PCa cell lines, compared to AR-positive ones (Fig. 3d). This result suggests that caspase-8 may be involved in NF-κB activation. To test this hypothesis, we focused once again on the modulation of caspase-8 expression. We observed that in the context of caspase-8 overexpression (LNCaP WT background), the phosphorylation levels of NF-κB and IκBα were increased (Fig. 3e). Moreover, the knockdown of caspase-8 in LNCaP ENZR cells led to decreased NF-κB signaling (Fig. 3f). This phenotype was also observed for original enzalutamide-resistant PC3 and DU145 cells with sh*CASP8* lenti-virus infection (Fig. S3b, c). Taken together, these results highlight a novel role of caspase-8 in NF-κB activation that in turn promotes ENZR.

To further dissect the relationship between caspase-8 and NF-κB in ENZR, we used Bay 11–7082 [24] to inhibit IκBα phosphorylation in the context of caspase-8 overexpression. The compound Bay 11–7082 inhibits the NF-κB signaling pathway as indicated by reducing phosphorylation of IκBα and consequent NF-κB(p65) phosphorylation, as well as preventing NF-κB(p65) nuclear translocation upon TNF-α stimulation (Fig. S3d-g). As shown in Fig. 3g, the significant effect of caspase-8 overexpression in cell survival under enzalutamide treatment (*P* < 0.0001, compared to luc-HA control) was abrogated by Bay 11–7082 treatment. This demonstrates that the blockage of NF-κB signaling rescues the sensitivity to enzalutamide in the context of caspase-8 overexpression and proves that the caspase-8-NF-κB axis is involved in ENZR.

### Caspase-8 scaffolding function regulates NF-κB signaling and the downstream secreted factors in ENZR

**Fig. 4:**
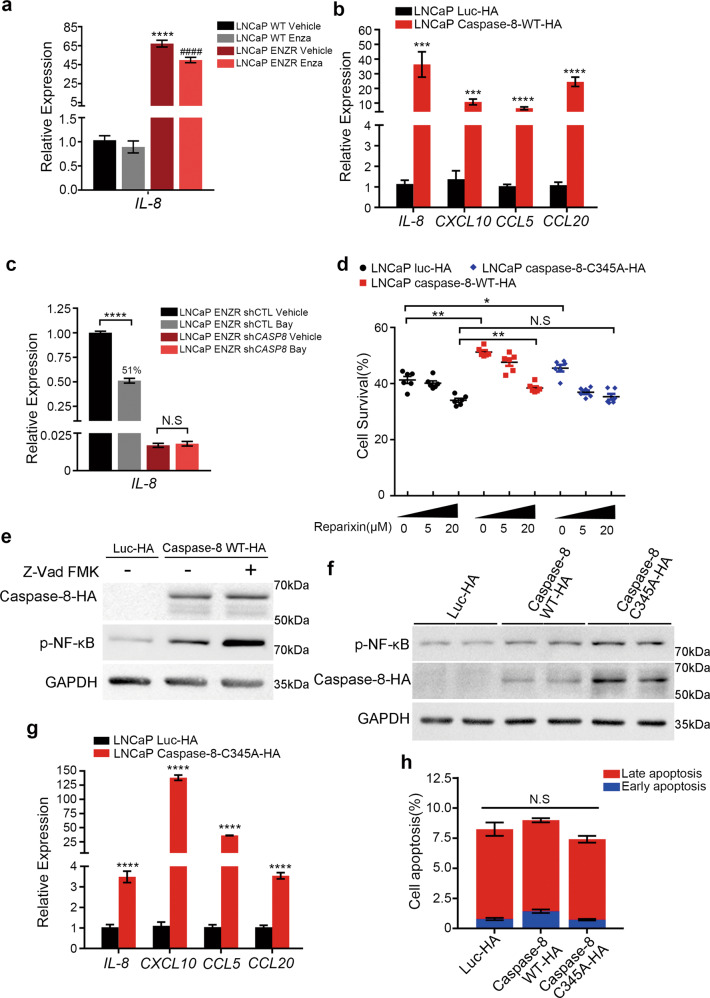
Caspase-8 scaffolding function dominantly regulates NF-κB pathway in enzalutamide resistance. **a** RT-qPCR analysis of *IL-8* relative expression in LNCaP WT and ENZR cells. Cells were treated with or without 10 μM enzalutamide for 48 h. *GAPDH* was used as an internal reference. *vs LNCaP WT vehicle, #*vs* LNCaP WT Enza. *n* = 9 from three independent experiments. **b** RT-qPCR analysis of *IL-8*, *CXCL10*, *CCL5*, *CCL20* relative expression in LNCaP luc-HA and LNCaP caspase-8-WT-HA. *GAPDH* was used as an internal reference. *n* = 9 from three independent experiments. **c** RT-qPCR analysis of *IL-8* relative expression in LNCaP ENZR shCTL and ENZR sh*CASP8* cells, cells were treated with 5 μM Bay 11–7082 for 24 h under serum starvation. *GAPDH* was used as an internal reference (*n* = 3). **d** Cell viability (%) of LNCaP overexpressing caspase-8 were analyzed under enzalutamide treatment and IL-8 signaling inhibition. 5 × 10^4^ of LNCaP caspase-8 WT-HA and LNCaP caspase-8 C345A-HA cells were seeded in six-well plates. After 24 h, adherent cells were treated with 10 μM enzalutamide or DMSO vehicle under the condition of 5, 20 μM Reparixin (inhibitor of IL-8 receptor) for 8 days supplemented with 2% FBS, cells were harvested and counted. Surviving cells were normalized to DMSO-treated control condition. LNCaP overexpressing luc-HA cells were used as a control. *n* = 6 from three independent experiments. **e** Western blot analysis protein level of caspase-8 and phosphorylation level of NF-κB in LNCaP cells overexpressed caspase-8-HA. Caspase-8-HA was transiently overexpressed in LNCaP cells by lentivirus. Culture medium supplemented with 20 μM Z-Vad FMK or DMSO vehicle were replaced 24 h after transfection. Cells were consequently harvested for western blot analysis after 72 h transfection. **f** Western blot analysis protein level of caspase-8 and phosphorylation level of NF-κB in LNCaP cells overexpressed caspase-8 WT-HA and caspase-8 C345A-HA (no catalytic activity mutation). **g** RT-qPCR analysis of *IL-8*, *CXCL10*, *CCL5*, *CCL20* relative expression in LNCaP luc-HA and LNCaP caspase-8 C345A-HA cells. *GAPDH* was used as an internal reference. *n* = 9 from three independent experiments. **h** Apoptosis analysis of LNCaP overexpressing caspase-8-WT-HA and caspase-8-C345A-HA. Cells were staining with FITC Annexin V and PI, and analyzed using flow cytometry. The percentage of early and late apoptosis cells were presented. LNCaP luc-HA cells were used as a control (*n* = 3).

Importantly, certain NF-κB downstream cytokines were involved in the progression of PCa [25]. Among our RNA-seq results, cytokines (e.g., *IL-8*, *CXCL10*, *CCL5*, *CCL20*), as well as IL-8-Receptor (*IL-8R*), *CXCR1*, and *CXCR2* mRNAs were significantly upregulated in LNCaP ENZR cells (Fig. S4a and Table S1). Importantly, all of these genes are NF-κB targets (Fig. 3a). qPCR analysis confirmed the significant elevation of *IL-8* in LNCaP ENZR and VCaP ENZR cells (Fig. 4a and Fig. S4b). Additionally, overexpression of caspase-8 in LNCaP WT resulted in increased mRNA levels of the abovementioned cytokines (Fig. 4b), while the knockdown of caspase-8 in LNCaP ENZR reduced expression of *IL-8* (Fig. 4c). In addition, Bay 11–7082 effectively inhibited *IL-8* expression in LNCaP ENZR, while not in LNCaP ENZR sh*CASP8* cells (Fig. 4c). More importantly, the pro-survival effect of caspase-8 under enzalutamide treatment was partially inhibited by reparixin, an IL-8R antagonist, demonstrating that IL-8 signaling was a downstream effector of caspase-8 in ENZR (Fig. 4d). Taken together, these results suggest that caspase-8 promotes ENZR via NF-κB activation and the consequent expression/secretion of inflammatory mediators.

Caspase-8 scaffolding function has been previously involved in NF-κB activation [15]. Interestingly, the use of a pan-caspase inhibitor, Z-Vad FMK, did not repress NF-κB activation triggered by transient caspase-8 overexpression (Fig. 4e). Consistently, the inhibition of caspase activity by Z-Vad FMK partially counteracted the effect of Bay 11–7082 treatment (*P* < 0.01) (Fig. 3g). Overall, these results suggest that the caspase-8 protease activity is not important for NF-κB activation.

We further explored NF-κB activation by caspase-8, using a caspase-8 C345A mutant, which retained its scaffolding function, but not its proteolytic activity. Similarly, to the effect observed for WT caspase-8, overexpression of caspase-8 C345A increased NF-κB activity and the downstream cytokine response in LNCaP WT (Fig. 4f-g). Moreover, the overexpression mutant caspase-8 in LNCaP WT still promoted ENZR (Fig. 4d, *P* < 0.05), unless IL-8 signaling was suppressed by reparixin treatment (Fig. 4d). Neither caspase-8 WT nor C435A stable expression led to apoptosis in LNCaP cells (Fig. 4h and Fig. S4c). Taken together, our results indicate that the scaffolding function of caspase-8 is the one important for NF-κB pathway activation and downstream cytokine responses in ENZR.

### cFLIP is important for caspase-8 pro-survival role

**Fig. 5:**
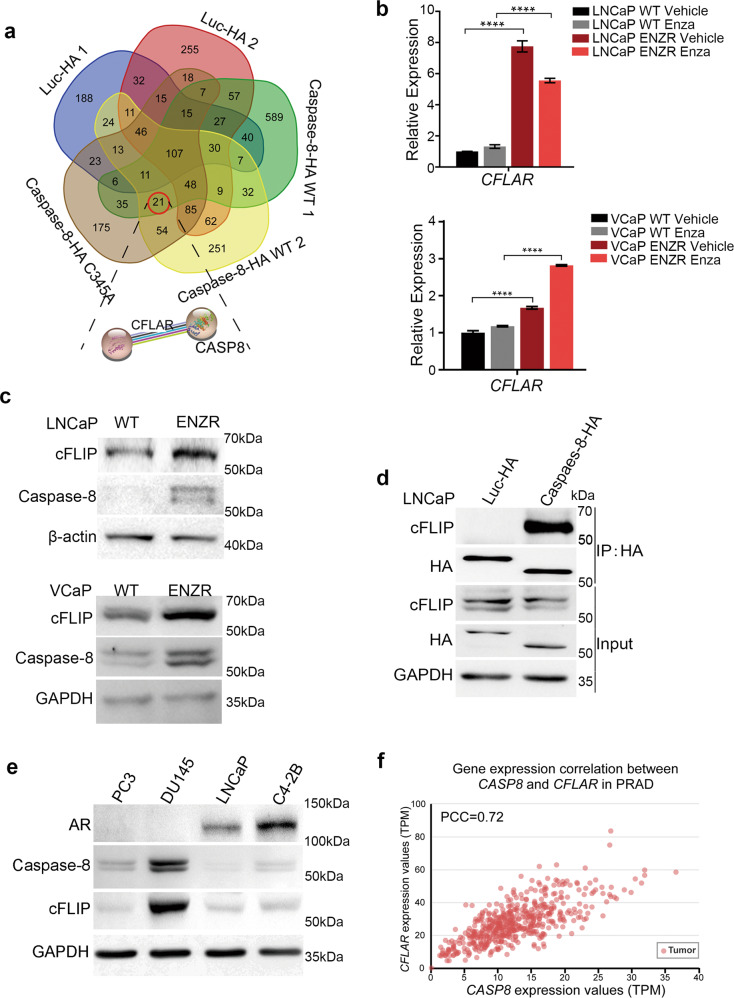
cFLIP is upregulated together with caspase-8 in PCa. **a** Venn diagram representing the proteins detected by IP-mass spectrometry. Two caspase-8-WT-HA samples, one caspase-8-C345A-HA sample and two luc-HA samples (as negative control) were evaluated. Twenty-one caspase-8-specific proteins were detected. Caspase-8-cFLIP interaction was analyzed by STRING and represented. **b** Relative mRNA level of *CFLAR* were analyzed by RT-qPCR under enzalutamide treatments for 48 h. LNCaP WT and LNCaP ENZR were treated with 10 μM enzalutamide or DMSO vehicle (top, *n* = 9 from three independent experiments). VCaP WT and VCaP ENZR were treated with 1 μM enzalutamide or DMSO vehicle (bottom, *n* = 3). *GAPDH* was used as an internal reference. **c** Western blot analysis protein level of caspase-8 and cFLIP in LNCaP WT and ENZR cells (top) and VCaP WT and ENZR cells (bottom). **d** LNCaP cells were stably expressed luc-HA and caspase-8-HA by lentivirus. Cells were lysed in MCLB buffer, proteins were subjected to IP using HA magnetic beads, and co-precipitation endogenous cFLIP was detected by Western blot. LNCaP luc-HA cells were used as a negative control. **e** Western blot analysis protein level of caspase-8, cFLIP, and AR in PC3, DU145, LNCaP, and C4-2B prostate cancer cells. **f** Correlation of *CASP8* and *CFLAR* gene expression in PRAD. The TPM of *CASP8* and *CFLAR* showed a strong correlation (PCC = 0.72).

To dissect the molecular mechanism mediates the switching of caspase-8 from pro-apoptotic to pro-survival role in ENZR, we identified protein interactions of caspase-8 using immunoprecipitation mass spectrometry (IP-MS) based proteomics analysis (Dataset S2). A total of 20 proteins were identified as caspase-8 high confidence interaction proteins (HCIPs) using a String analysis (Fig. 5a and Fig. S5a). A reactome analysis revealed that several HCIPs were enriched in caspase-8-dependent cell death and survival functions including *TRAIL signal*, *CASP8 activity is inhibited*, etc. (Fig. S5b). Among the HCIPs, *CFLAR* (also named as cFLIP) has the one showing the highest transcription level fold change in LNCaP ENZR compared to LNCaP WT (Table S1). This finding was further confirmed by qPCR and western blot in both the LNCaP and VCaP ENZR cells (Fig. 5b, c). cFLIP is an enzymatically inactive homolog of caspase-8 [26]. The interaction of exogenous caspase-8 and cFLIP was validated in both the LNCaP and 293 T cells by co-immunoprecipitation (Fig. 5d and Fig. S5c). Interestingly, among all the PCa cell lines, DU145 was the one showing the highest cFLIP protein levels, and also the highest caspase-8 expression (Fig. 5e). Moreover, we found that there was a high correlation between *CASP8* and *CFLAR* transcriptional expression in PCa patients (data retrieved from TCGA database; Fig. 5f). These results overall suggest that cFLIP is closely connected with caspase-8 in PCa.

**Fig. 6:**
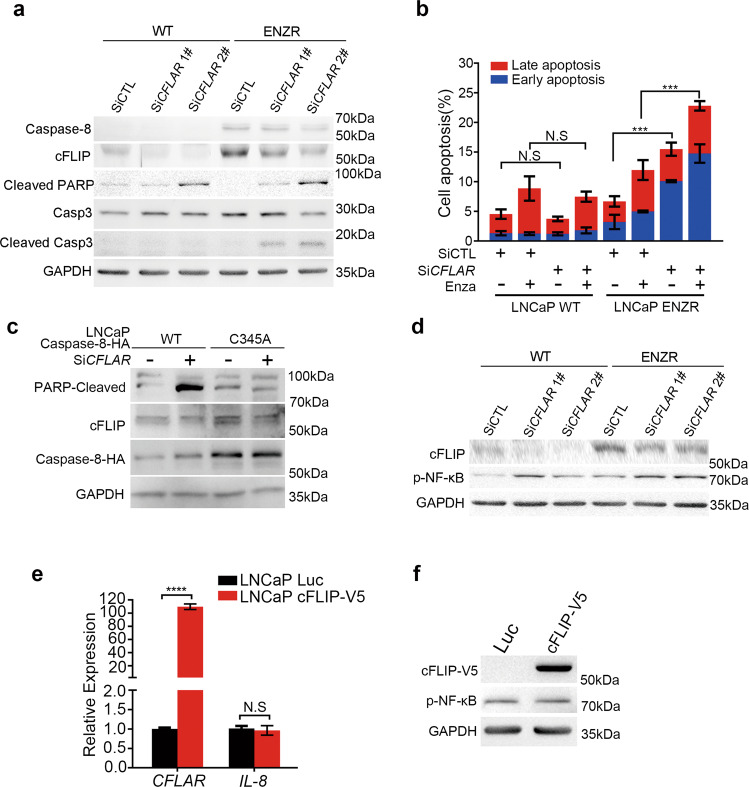
cFLIP inhibits the caspase-8 pro-apoptotic potential in ENZR. **a** Western blot analysis protein level of caspase-8, cFLIP, caspase-3, cleaved-caspase-3, and cleaved PARP under the condition of LNCaP WT and LNCaP ENZR cells were transfected with 100 nM si*CFLAR* 1^#^ and si*CFLAR* 2^#^ for 3 days. **b** Apoptosis analysis of the effect of si*CFLAR* in LNCaP WT and ENZR cells using flow cytometry. Cells were treated with 100 nM si*CFLAR* 2^#^ and cultured for 3 days supplemented with 25 μM enzalutamide or DMSO vehicle. Then, cells were harvested, stained with FITC Annexin V and PI and analyzed using flow cytometry. The relative percentage (%) of early and late apoptosis cells were presented. Scramble siCTL was used as a negative control (*n* = 3). **c** Western blot analysis protein level of caspase-8, cFLIP, and cleaved PARP, under the condition of LNCaP caspase-8 WT-HA and caspase-8 C345A-HA cells were transfected with 100 nM si*CFLAR* for 3 days. **d** Western blot analysis protein level of cFLIP and phosphorylation level of NF-κB cleaved PARP under the condition of LNCaP WT and LNCaP ENZR cells were transfected with 100 nM si*CFLAR* 1^#^ and si*CFLAR* 2^#^ for 3 days. **e** RT-qPCR analysis of *IL-8* relative expression in LNCaP cells which stably overexpressing luc and cFLIP-V5. *GAPDH* was used as an internal reference. *n* = 9 from three independent experiments. **f** Western blot analysis protein level of cFLIP and phosphorylation level of NF-κB in LNCaP cells which stably overexpressing luc and cFLIP-V5 under serum starvation for 24 h.

We then tested whether cFLIP could be involved in caspase-8 pro-survival role. Transient knockdown of cFLIP dramatically induced apoptosis and reduced cell growth in LNCaP ENZR, but not in LNCaP WT, with elevated cleaved-caspase-3 and cleaved PARP, downstream of caspase-8 in apoptosis cascade (Fig. 6a), phenocopying the condition of excessive caspase-8 overexpression in LNCaP WT cells (Fig. S2c). Flow cytometry results further confirmed the induction of apoptosis upon cFLIP silencing in LNCaP ENZR but not LNCaP WT (Fig. 6b and Fig. S5d, e). In contrast, in caspase-8 C345A stable overexpressed LNCaP WT, silencing of cFLIP did not induce apoptosis as determined by PARP cleavage, while in caspase-8-WT stable overexpressed cells did (Fig. 6c). These results suggest that cFLIP inhibits the pro-apoptotic function of caspase-8 probably via specific binding events. However, irrespectively of caspase-8 overexpression, NF-κB phosphorylation was mildly upregulated upon cFLIP knockdown in LNCaP cells (Fig. 6d). On the other hand, the cFLIP overexpression did not influence NF-κB phosphorylation or *IL-8* mRNA levels in LNCaP WT cells (Fig. 6e-f). These results indicated that the high expression of caspase-8, rather than cFLIP, plays a central role in NF-κB activation and ENZR. Collectively, our results suggest that cFLIP inhibits the caspase-8 apoptotic role, promoting its pro-survival function in PCa cells resistant to enzalutamide.

### Caspase-8 levels correlate positively with prostate cancer clinical progression

**Fig. 7:**
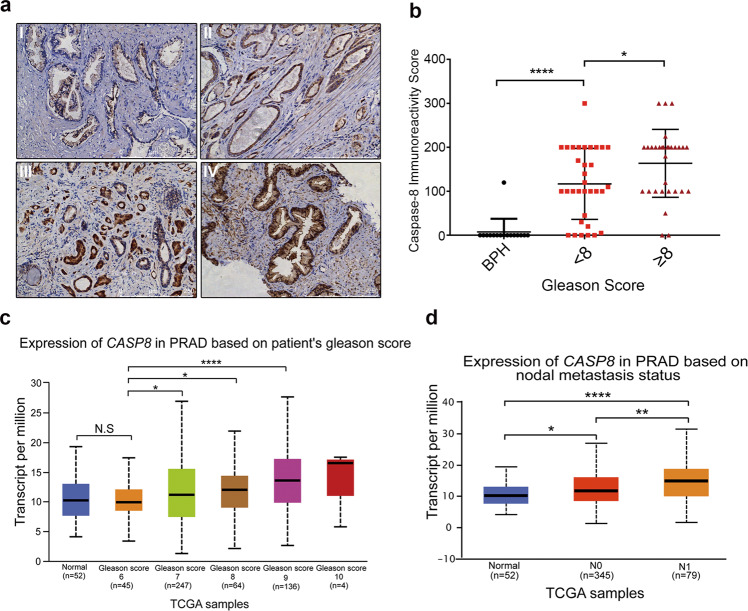
Caspase-8 levels correlate positively with prostate cancer clinical progression. **a** Representative image of immunohistochemical analyses of caspase-8 in benign prostate gland and prostate cancer tissues. (I, BPH; II, GS: 4 + 3; III, GS: 3 + 4; IV, GS:5 + 4), scale bar: 250 μm. **b** Quantitative IHC expression of caspase-8 scored by immunoreactivity score in BPH, low/intermediate-risk PCa groups (GS < 8), and high-risk PCa groups (GS ≥ 8). **c** Caspase-8 transcriptional levels in PRAD with different Gleason scores. Caspase-8 was remarkably upregulated in patients with higher Gleason scores in TCGA expression data. **d** Caspase-8 transcriptional levels in PRAD with different nodal metastasis status. Caspase-8 was remarkably upregulated in more aggressive nodal metastasis in TCGA dataset.

To determine the significance of caspase-8 in PCa progression, we first investigated the caspase-8 expression levels in PCa tissues using TCGA database from UALCAN resource [27]. Caspase-8 mRNA was significantly upregulated in primary prostate adenocarcinoma, compared to normal prostate tissue (*P* < 0.01) (Fig. S6a). Further, we compared the expression of caspase-8 in 16 benign prostate hyperplasia (BPH) tissues and 63 PCa tissues using immunohistochemistry (IHC). A representative picture of caspase-8 expression is shown in Fig. 7a. Faint cytoplasmic immunostaining for pro-caspase-8 was present in the majority of BPH samples (93.8%), while 56 in total 63 of PCa samples (88.8%) presented positive (Fig. 7b). The immunoreactivity score indicated the significant higher expression of cytoplasmic caspase-8 in tumor samples than in BPH tissues (*P* < 0.0001). Two groups of PCa (GS < 8; GS ≥ 8) were defined according to NCCN (National Comprehensive Cancer Network) guidelines [28], which related to low/intermediate and high potential clinical risk. Cytoplasmic caspase-8 was significantly higher in high-risk group than in low/intermediate-risk groups (*P* < 0.05, Fig. 7b). Similarly, TCGA database supported the positive correlation of caspase-8 expression and Gleason Score (GS). Patients who were classified as intermediate or high risk (GS ≥ 7) expressed significantly higher caspase-8, compared to low-risk patients (GS = 6) (GS 6 *vs* GS 7,8: *P* < 0.05; GS 6 *vs* GS 9: *P* < 0.0001, Fig. 7c). Moreover, patients with nodal metastasis showed remarkably higher caspase-8 expression levels (Normal *vs* N_0_: *P* < 0.05; N_0_ *vs* N_1_: *P* < 0.01, Fig. 7d). Interestingly, 81.8% (9/11) nuclear positive were detected in high caspase-8 group (Table S2 and Fig. S6b) in PCa samples, which was consist with the recent study of the correlation between the nuclear caspase-8 and tumor treatment resistance [29].

## Discussion

**Fig. 8:**
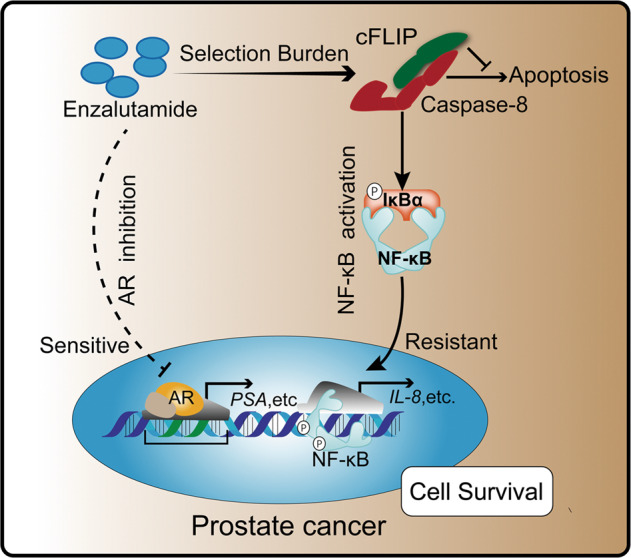
The role of caspase-8 in enzalutamide resistance. Enzalutamide inhibits prostate cancer progression by blocking androgen receptors. However, the selection burden of long-term enzalutamide treatment induces caspase-8 high expression. Caspase-8 promotes cell survival under enzalutamide treatment via NF-κB activation and the consequent expression of IL-8 and other NF-κB target genes.

In this study, we revealed an unanticipated role of caspase-8 in ENZR. We discovered that caspase-8 scaffolding function promotes NF-κB activation and consequently IL-8 expression. Under enzalutamide treatment, this caspase-8-specific pro-survival function requires the interaction with cFLIP, and is independent of its proteolytic activity (Fig. 8).

Avoiding apoptosis is one of the hallmarks of cancer. As a central player in the initiation of apoptotic cascade, caspase-8 expression [30] or its activity [31] are often reduced in cancer. In our study, both patient samples and TCGA database-based analysis point to a positive correlation between caspase-8 and PCa poor prognosis. In fact, in many tumors, caspase-8 expression is retained or even increased [32], raising the question whether caspase-8 promotes tumor development and progression under certain circumstances. Several non-canonical functions of caspase-8 including modulating cell adhesion and migration have been identified in cellular studies [33]. NF-κB activation was proposed as a pro-survival alternative function of caspase-8 in cancer. Specifically, G325A mutation was showed to activate NF-κB signaling to a greater extent in head and neck squamous cell carcinoma [34]. Furthermore, caspase-8 expression enhanced the resistance to temozolomide via NF-κB activation in glioblastoma cells [35]. Our study, aligns with these findings, demonstrating the role of caspase-8 in NF-κB activation, promoting PCa survival [36]. Previous studies did not explore how caspase-8 and its functions regulated, particularly the determinant which switches caspase-8-mediated essential effects from pro-apoptosis to pro-survival to promote tumor growth remained unknown [37].

Some studies found that caspase-8-mediated NF-κB activation was dependent [38], while others reported it was independent of its enzymatic activity [39]. Our results support the notion that the scaffolding function of caspase-8 is necessary for NF-κB activation, but how this happens at the molecular level, remains unclear. Specially, we found that caspase-8-C345A catalytical-inactive mutant also increased NF-κB phosphorylation, comparably to caspase-8-WT. However, it is worth noticing that caspase-8-WT overexpression increased IL-8 expression and cell viability in a greater extent than that of catalytical-inactive mutant one. One possible explanation for this phenomenon is a minor role of caspase-8 enzymatic activity in NF-κB regulation. The ambiguous enzymatic function of caspase-8 needs to be clarified in future studies. IL-8 is a pro-angiogenic cytokine associated with PCa aggressiveness and poor prognosis [25]. How IL-8 in PCa is regulated remains unknown. It is widely accepted that constitutively activated NF-κB signaling correlates with higher GS and poor progression in PCa [40]. Our findings further revealed a causal link between caspase-8/NF-κB/IL-8 and the acquisition of ENZR.

Our study revealed an interesting interaction between the cFLIP and caspase-8. cFLIP is a caspase-8 homolog lacking proteolytic activity [26], one can be partially cleaved by pro-caspase-8, generating p12, and p43 subunits. Caspase-8 was demonstrated to dimerize with p43 [41], but whether the resulting heterodimers have pro- or antiapoptotic functions remained controversial [42–44]. Elevation of cFLIP has been previously reported in CRPC, antagonizing the response to AR-targeted therapy [45]. Interestingly, in enzalutamide-resistant PCa cell lines (DU145, LNCaP ENZR, and VCaP ENZR), both caspase-8 and cFLIP were highly expressed. Furthermore, we observed that both wide-type and catalytical-inactive mutant caspase-8 could bind to cFLIP in PCa cells. Moreover, cFLIP knockdown resulted in caspase-8 mediated apoptosis in PCa, supporting the antiapoptotic function of cFLIP/caspase-8 heterodimers.

cFLIP was reported to inhibit FADD-Caspase-8-RIPK1 complex-mediated NF-κB activation [46]. In opposition, others showed that p43 of cFLIP promoted NF-κB activation [47]. Here, we observed that cFLIP did not significantly activate NF-κB signaling, indicating caspase-8-mediated NF-κB activation may be regulated by others. We identified both FADD and cFLIP in our caspase-8-HA WT and mutant interactomes, in line with the previous findings [48]. Interestingly, the caspase-8 scaffolding function has been related to several protein complexes and the enhancement of downstream cytokine/chemokine secretion [49].

In our study, we found that PC3 and DU145 showed constitutive active NF-κB, contrarily to AR-positive LNCaP WT and C4-2B cells. Therefore, it is possible to speculate that AR loss or inhibition and constitutive NF-κB activation may be correlatable events. In this case, NF-κB activity could be necessary for sustaining PCa cell survival and growth following AR loss [17]. It was reported that caspase-8 repressed AR-dependent gene expression in the present of androgen [50]. However, we did not observe significant changes of AR and its target genes’ expression, upon caspase-8 overexpressing or silencing (under low androgen levels). This indicates that caspase-8 promotes ENZR independently of AR. While we observed the significant upregulation of AR and its downstream genes in ENZR cells, further studies are needed to explain how AR expression is upregulated upon continuous enzalutamide treatment.

As contributors to PCa heterogeneity, tumor cells that are strongly dependent on NF-κB signaling and cytokine production, but not on the AR pathway, may have a selective advantage in retaining caspase-8 expression. Thus, our findings have important implications for the consideration of enzalutamide therapy, particularly if considered for long periods of time. Further studies are, however, needed to test whether the combination of caspase-8/NF-κB/IL-8 inhibitors and enzalutamide may improve the treatment efficacy and outcomes in CRPC.

## Materials and methods

### Tissue collection and ethical approval

A total of 79 pathological tissues from patients with prostate cancer or benign prostate hyperplasia were collected at Xiangya Hospital (Changsha, China) from Jan 2018 to Jan 2020. All postoperative pathological tissue from patients were confirmed PCa or BPH as defined by a trained pathologist. Our study was approved by the Ethics Committee of Xiangya School of Medicine, Central South University (Registration number: #202012234). Written informed consents were obtained from all patients.

### Cell lines and reagents

LNCaP, PC3, DU145, C4-2B cell lines were originally acquired from ATCC. 22Rv1 cell line was provided by Stem Cell Bank, Chinese Academy of Sciences. VCaP WT and VCaP ENZR were kindly provided from Lab Zhenfei Li, Shanghai Institute of Biochemistry and Cell Biology, Chinese Academy of Sciences. LNCaP, PC3, DU145, C4-2B, and 22Rv1 were cultured in RPMI 1640 (HyClone) with 10% fetal bovine serum (FBS; Gibco). 293 T and VCaP were cultured in DMEM/High Glucose (HyClone) with 10% FBS at 37 °C with 5% CO_2_. All cell lines were tested mycoplasma negative using DAPI staining. Enzalutamide, Z-Vad-FMK, Bay 11–7082, and Reparixin inhibitors were purchased from ApexBio. Enzalutamide (100 mM), Z-Vad-FMK (50 mM), Bay 11–7082(50 mM), and Reparixin (40 mM) were suspended in DMSO. hTNF-α (200 μg/ml) was purchased from Biolegend. All antibodies used in were list in Table S3.

### Plasmid construction and transfection

Lentivirus vector pLL3.7 was a gift from Luk Parijs (Addgene #11795) [51]. ShRNA sequences were subcloned into PLL3.7 for stably knockdown of caspase-8 and cFLIP, respectively. cDNAs encoding h*CASP8* and h*CFLAR* were subcloned into pHAGE-C-HA-Puro lentivirus vector [52] for caspase-8-HA or pLenti CMV-C-V5-neo lentivirus vector for cFLIP-v5 overexpression. For stable transfection, lentivirus was generated by transfection with the target or negative control vectors using packaging plasmid mix (Tet2 + MPMG + Rev + VSV-G) with polyethyleneimine (PEI, Polyscience) into 293 T cells. Pseudo-virus was harvested 48 h later, filtered and used to infect cells with 10 μg/mL polybrene. Infected cells were screened using 1 μg/ml puromycin for 3 days or 500 μg/ml G418 for 6 days. For transient transfection, all siRNA were synthesized from Sangon Biotech. siRNA (50–100 nM) transfection was performed using lipofectamine 2000 (Thermo Scientific) and Opti-MEM (Gibco) according to the manufacturer’s protocols. Cells were harvested 3–4 days after transfection. All shRNA/SiRNA used in this study were list in Table S4.

### Generation of LNCaP Enzalutamide-Resistant (LNCaP ENZR) cell lines

The generation of LNCaP ENZR was performed as previously described [23]. Briefly, LNCaP cells were seeded and sub-cultured in the presence of enzalutamide for about 6 months and authenticated by short tandem repeat (STR) profiling (Biowing) before the downstream experiment. Briefly, the concentration of enzalutamide was progressively increased from 1 to 10 μM. The parental LNCaP cell line was sub-cultured in parallel with DMSO vehicle. LNCaP ENZR was passaged every 5–7 days and maintained in a growth medium supplemented with enzalutamide for 3–5 days every passage. After 6-month culture, the IC_50_ of enzalutamide was measured to confirm the successful generation of LNCaP ENZR.

### Enzalutamide sensitivity determination

1 × 10^3^ LNCaP WT, LNCaP ENZR, PC3, and 22Rv1 were plated on 96-well plates and left to adhere for 24 h. Cells were then treated with enzalutamide in a growth medium, and maintained for 5–7 days at 37 °C with 5% CO_2_. 2 × 10^4^ VCaP WT and ENZR were plated and consequently treated with enzalutamide for 4 days at 37 °C with 5% CO_2_ as described above. Cell viability was analyzed using CCK-8 assay (ApexBio). Absorbance was measured at OD450 nm (BioTek) and recorded as normalized values to drug-free condition. The IC_50_ of each cell lines were calculated using GraphPad Prism.

LNCaP caspase-8-HA and luc-HA cells were plated in six-well plates at a density of 5 × 10^4^ cells per well and allowed to adhere for 24 h. Cells were refreshed to growth medium supplemented with 2% FBS and inhibitors: Enzalutamide (10 μM), Z-Vad-FMK (20 μM), Bay 11–7082 (1 μM) and Reparixin (5, 20 μM), or DMSO vehicle and then incubated for another 7–9 days. The growth medium were refresh every 3 days. In final, cells were digested and counted using a cell counter (JIMBIO FIL). The viability was calculated as the abovementioned.

### Clonogenic assays

Cells were seeded in 6-well plates with 2 × 10^3^ cells per well and incubated in the growth medium supplemented with enzalutamide or DMSO vehicle for 14 days with medium changed every 4–5 days. In final, cells were fixed with 4% PFA, stained with 0.1% crystal violet. The numbers of colonies were scanned and counted using Image J. 1.50b.

### Microarray data and RNA-Seq

Enzalutamide-resistance-related datasets were retrieved from the GEO database (https://www.ncbi.nlm.nih.gov/geo/; accession numbers GSE44927 and GSE69896). For RNA-seq, total RNA was isolated using the RNeasy mini kit (Qiagen). Library construction and sequencing were performed at Shanghai Sinomics Corporation. Briefly, paired-end libraries were synthesized using TruSeq™ RNA Sample Preparation Kit (Illumina), according to manufacturer’s instructions. Purified libraries were quantified in the Qubit® 2.0 Fluorometer (Life Technologies) and validated using Agilent 2100 bioanalyzer (Agilent Technologies). Clonal clusters were generated by cBot and sequenced on the Illumina NovaSeq 6000 platform (Illumina). Fastq files were trimmed using FastQC to ensure the quality of the reads. Clean reads were mapped to GRCh38.91 with the Hisat2 tool. Mapped reads were transferred to FPKM (number of kilobases per million mapped fragments of the exon model) to characterize the expression of the genes comprehensively. The obtained FPKM values were used for horizontal comparison between different samples and groups by software edgeR [53]. Genes with FPKM ≥ 1 were extracted for further pathway and network analyses.

### Real-Time PCR

Total RNA was isolated from prepared cell samples using Trizol reagent (TianGen).1ug total RNA was reverse transcript into cDNAs using HiScript III RT SuperMix (Vazyme) for each reaction according to manufacturers’ instructions. 1/100-1/20 of each reaction cDNAs per 384-plate well were used for real-time PCR in LightCycler480 apparatus (Roche) using SYBR Green Master Mix (Yeasen). Primers used for real-time PCR were listed in Table S5. *GAPDH* was used as the internal control gene. Relative gene expression data were analyzed using the 2^-ΔΔCT^ method.

### Extraction of protein and western blotting

Cells were washed with cold DPBS twice and lysed using Mammalian Cell Lysis Buffer (MCLB): 50 mM Tris pH 7.5, 150 mM NaCl, 0.5% NP40, supplemented with phosphatase inhibitor cocktail (Roche), 1 mM PMSF (Amresco), and complete EDTA-free protease inhibitor cocktail (Roche). Protein amounts were quantified by the Bradford assay (Bio-Rad) and the same quantity from each sample was electrophoresed by SDS PAGE using vertical electrophoresis (BioTanon). Proteins were then transferred to nitrocellulose blotting membranes (Milipore) using electrophoretic transfer apparatus (BioTanon). Membranes were blocked in 5% nonfat milk incubated with suitable primary overnight at 4 °C and subsequently incubated with secondary antibodies (1:10,000 diluted in TBST) at room temperature for 40 min. Images were obtained using the ChampChemi Imaging System (Sage Creation Science). GAPDH or β-actin were used as internal control proteins.

### NF-κB activation detection

For detecting the constitutive activation of NF-κB pathway with modified caspase-8 expression. LNCaP cells were treated with inhibitors (Enzalutamide and/or Bay 11–7082) and serum starved for 24 h. Cells were harvested using MCLB, supplemented with phosphatase inhibitor cocktail (Roche), protease inhibitor cocktail (Roche) and 1 mM PMSF to gain the whole-cell protein lysis. Phoso-NF-κB or IκBα were detected. For detecting the inhibit efficiency of Bay 11–7082 on NF-κB pathway, LNCaP cells were preincubated in growth medium supplemented with 2% FBS and Bay 11–7082 for 30 min, then cells were treated with 15 ng/ml hTNF-α for 15 min or 60 min. Cells were harvested as mentioned above.

### Immunoprecipitation and mass spectrometry

293 T cells were lysed at 4 °C in MCLB, supplemented with 1 mM PMSF and complete EDTA-free protease inhibitor cocktail. Proteins in supernatant were separated using centrifugation (4 °C, 12,000*×g*) for 20 min. Supernatant fractions were saved as loading input controls. Supernatant were subjected to immunoprecipitation with HA magnetic beads (Thermo Scientific) overnight at 4 °C. Beads were then washed 5 times in lysis buffer and then 3 times in DPBS. Binding proteins were eluted with a 0.1 M glycine solution, pH 2.0. Partially eluted samples were pre-processed and injected to an LTQ-Orbitrap Fusion Mass Spectrometry (Thermo Scientific) coupled with EASY-nLC 1000 Liquid Chromatograph Instrument (Thermo Scientific). Bioinformatics and statistical analysis of original mass spectrometric data were performed using Peaks Studio based on UniProt database (20180524, 20349).

### Flow cytometry

Apoptosis was detected via flow cytometry (BD Biosciences) using an Annexin V-FITC/PI apoptosis detection kit (Yeasen) according to the manufacturer’s instructions.

### Immunofluorescence

Treated cells were washed with DPBS twice and fixed with 4% paraformaldehyde (PFA) at room temperature for 15 min. After that, cells were permeabilized with 0.2% TritonX-100 for 8 min, washed with PBS for 5 min, blocked in 2% goat serum and 2% BSA/PBS for 1 h, incubated with rabbit NF-κB(P65) antibody, then with Alexa Fluor rabbit 568-conjugated secondary antibody, and then stained with DAPI. The cell slides were observed under a Leica confocal microscope.

### Immunohistochemistry staining

5 um duplicated slides cut from formalin-fixed and puffin-embedded PCa tissues as well as BPH tissues were obtained from the pathology department of Xiangya Hospital. All patients were confirmed PCa or BPH as defined by a trained pathologist. All sections were deparaffinized followed by heat-induced antigen retrieval in an autoclave in acetate citrate buffer, pH 6.0 for 5 min. Primary Rabbit-pro-caspase-8 antibody (Abcam) was used in a final solution of 1:500. Secondary antibody was applied for 30 min at 37 °C, and color was developed with a diaminobenzidine peroxidase substrate kit (Impact DAB, Vector Laboratories). Sections were then counterstained with hematoxylin, dehydrated, and mounted. Caspase-8 expression was visualized utilizing the Envision System (DAKO).

### Immunohistochemical analysis

Nuclear and cytoplasmic staining of Caspase-8 was evaluated separately for each sample and quantified as described previously [54]. In brief, staining intensity (negative = 0, weak = 1 + , moderate = 2 + , strong = 3 + ) and fraction of positive tumor cells were recorded for each tissue slides. The two parameters were multiplied to obtain an immunoreactivity score ranging from 0 to 300. A final score was built according to the following scores: Negative scores (0–5), weak scores (6–100), moderate scores (101–200), and strong scores (201–300). Negative and weak scores were defined as low caspase-8 group, moderate and strong scores were defined as the high one. Each stained slide was evaluated by two experienced pathologists independently.

### Pathway and network analyses

For RNA seq data, DEGs were the ones with a *P* value < 0.05 and a FC (Fold Change) > 1.5. DEGs of LNCaP WT and ENZR cells were uploaded into the IPA (Ingenuity Pathway Analysis) software (Qiagen) to perform network analysis. Interaction networks were built using the IPA building pathway function, based on the Ingenuity Knowledge Base. Cytoscape (V3.5.1) was used to draw the networks.

Gene expression changes related to biologically enriched pathways were screened using Gene Set Enrichment Analysis (GSEA) V4.0.3. DEGs were screened in the collections H (Hallmark Gene Sets) database to identify signatures. The GSEA plot, normalized enrichment score (NES), and FDR were derived from GSEA output, and the Hallmark signatures were ranked according to NES.

For IP-MS results, protein accession lists of two caspase-8-WT-HA and one caspase-8-C345A-HA samples were overlapped with the ones from two extra luc-HA IP-MS lists to exclude non-specific protein-protein interactions. Caspase-8-specific binding proteins were added to STRING V11.1 (https: //string-db.org/) to build the protein networks.

### Clinical data

Clinical data were retrieved from the TCGA database (level 3). The UALCAN tool (http://ualcan.path.uab.edu) was used to perform deep RNA-seq analyses. We analyzed the relative transcriptional expression of candidate genes in prostate cancer divided by relative clinicopathologic parameters, based on sample types (normal *vs* tumor tissues), nodal metastasis status, and Gleason score. Gene expression correlations between *CASP8, CFLAR, and NFKB1* in prostate cancer were analyzed using the Pearson correlation coefficient.

### Statistical and image analyses

Data were analyzed using GraphPad Prism software V7.04. All data are shown as Mean ± SEM for experiments performed with at least three replicates. The normal distribution was measured by D’Agostino-Pearson omnibus normality test. For measurement data, a two-tailed independent Student’s *t* test was conducted. Statistical significance for clinicopathological parameters were calculated using Chi-square test or Chi-square with Yates’ correction. Statistical significance was denoted as follows: ****p < 0.0001; ***p < 0.001; **p < 0.01; *p < 0.05; N.S No Significant.

## Supplementary information

Supplemental Dataset 2

Supplemental Dataset 1

Supplementary figures and tables

## Data Availability

Data required to support the findings of this study are present in main text or supplementary materials. All other data supporting the findings of this study are available from the corresponding author upon reasonable request.
