# Supplementary material for: Non-apoptotic function of caspase-8 confers prostate cancer enzalutamide resistance via NF-κB activation
Source: Cell Death Dis. 2021 Sep 4;12(9):833. doi: 10.1038/s41419-021-04126-4 (PMC8418603; doi:10.1038/s41419-021-04126-4)
Supplement: Supplementary file 3 — Supplementary figures and tables [file 41419_2021_4126_MOESM3_ESM.docx]

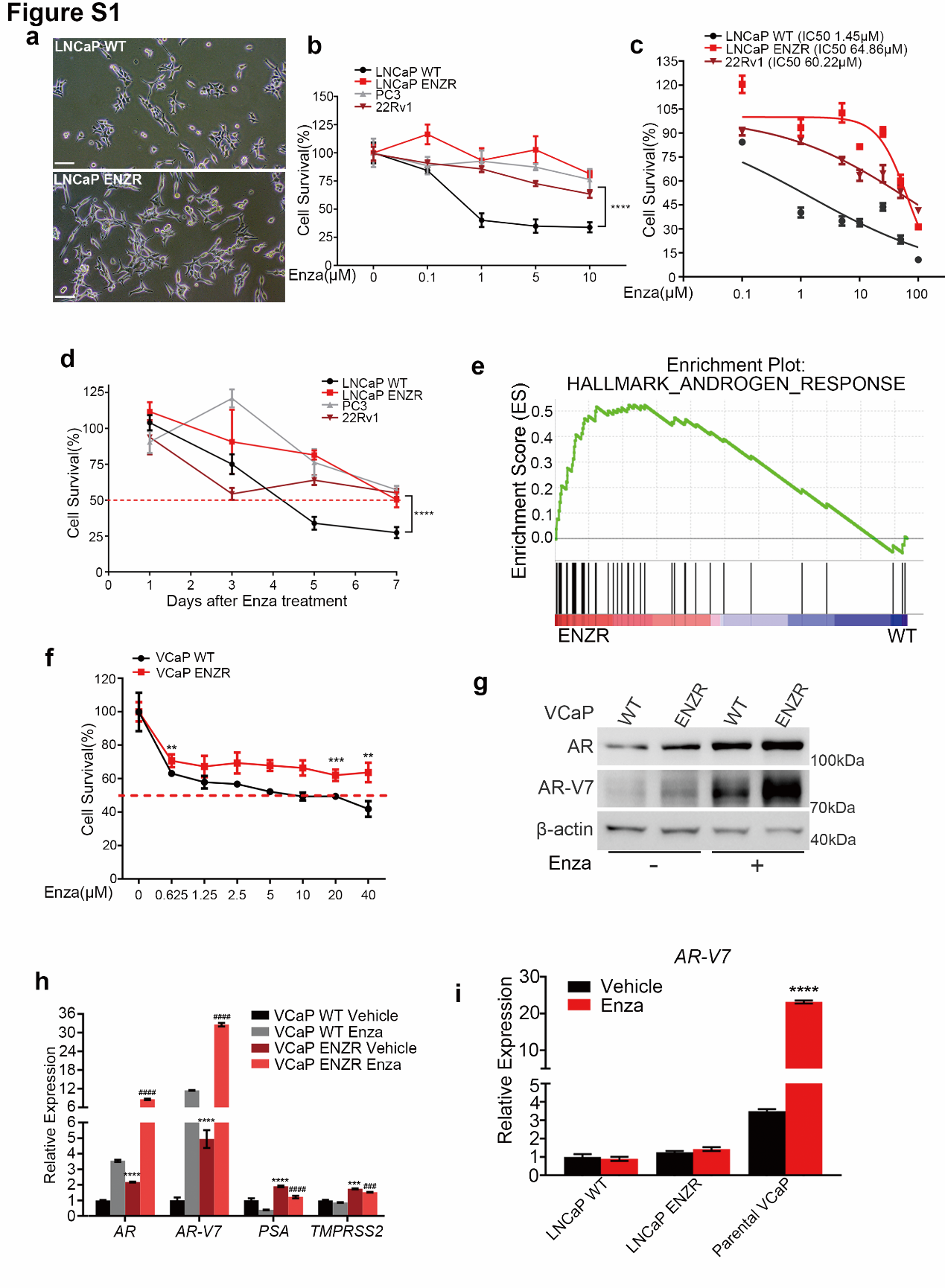


**Supplementary Fig. 1.**

**The characteristic of enzalutamide-resistance prostate cancer cell lines**

**a** Morphology of LNCaP WT and ENZR cells in bright field. Scale bar: 50 μm.

**b** Cell survival curve for prostate cancer cells treated with enzalutamide. Briefly, 1×10^3^ prostate cancer cells (LNCaP WT, LNCaP ENZR, PC3 and 22Rv1) were seeded in 96-well plates and after 24 h, adherent cells were treated with growth medium supplemented with indicated concentrations of enzalutamide. After 5-day treatment, cell viability was analyzed using CCK-8 and normalized to the drug-free condition (n = 3).

**c** The IC_50_ curve of LNCaP WT, LNCaP ENZR and 22Rv1 treated with enzalutamide for 5 days as above mentioned. The IC_50_ of LNCaP WT, LNCaP ENZR and 22Rv1 was 1.45 μM, 64.86 μM and 60.22 μM, respectively (n = 3).

**d** Cell survival curve for prostate cancer cells treated with enzalutamide. Briefly, 1×10^3^ prostate cancer cells (LNCaP WT, LNCaP ENZR, PC3 and 22Rv1) were seeded in 96-well plates and after 24 h, adherent cells were treated with growth medium supplemented with indicated concentrations of enzalutamide. Cell viability was analyzed every 48 h after treatment for 7 days using CCK-8 and normalized to the drug-free condition (n = 3).

**e** Gene Set Enrichment Analysis of DEGs profile in LNCaP WT and ENZR cells against Hallmark androgen response. The NES was 3.022 and FDR q-value < 1*10^-5^.

**f** Cell survival of VCaP ENZR treated with enzalutamide. 1.5×10^4^ VCaP WT and ENZR were seeded in 96-well plates. 24 h later, cells were treated with indicated concentration of enzalutamide. 3 days after enzalutamide treatment, cell viability was analyzed using CCK-8 and normalized to the drug-free condition (n = 3).

**g** Western blot analysis protein level of AR and AR-V7 in VCaP WT and VCaP ENZR cells under the treatment of 1μM enzalutamide for 48h.

**h** RT-qPCR analysis of AR, AR-V7 and AR target genes (PSA, TMPRSS2) relative expression in VCaP WT and VCaP ENZR cells under the treatment of 1μM enzalutamide for 48 h. GAPDH was used as internal reference. * vs. VCaP WT vehicle, # vs. VNCaP WT Enza (n = 3).

**i** RT-qPCR analysis of AR-V7 relative expression in LNCaP WT, LNCaP ENZR and parental VCaP. GAPDH was used as internal reference (n = 3).

Data are represented as Mean ± SEM. Statistical relevance was assessed using the Student’s t-test and is represented: * P < 0.05; ** P < 0.01; *** P < 0.001; ****P < 0.0001; N.S No significant.


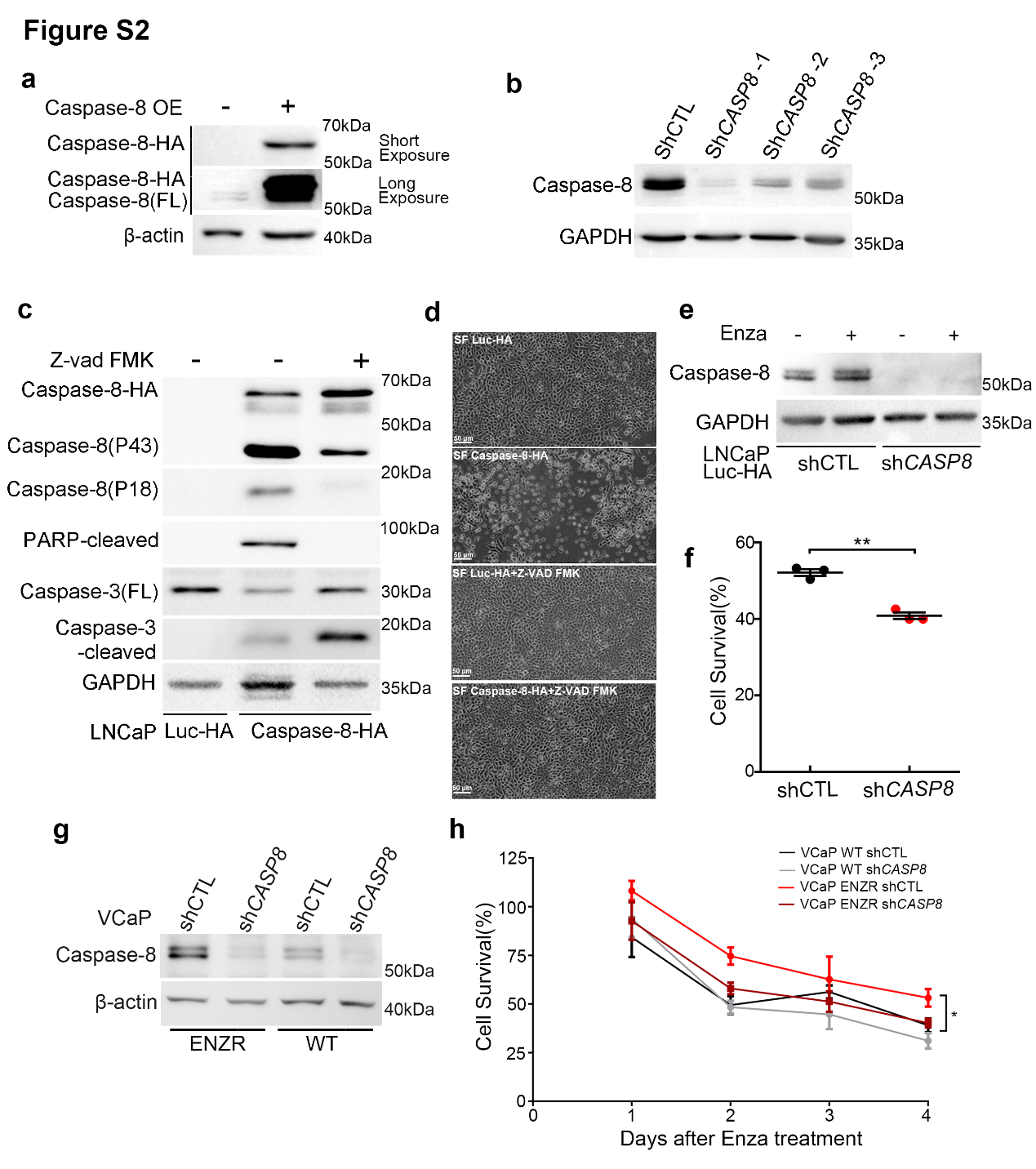


**Supplementary Fig. 2.**

**The role of caspase-8 expression in prostate cancer cells**

**a** Western blot analysis protein level of caspase-8 in LNCaP overexpressing caspase-8-HA and luc-HA cells.

**b** Western blot analysis protein level of caspase-8 in LNCaP ENZR sh*CASP8* cell lines. Lentivirus- RNAi-vector pll3.7 was used to construct the cell lines. The sh*CASP8*-1 showed the highest efficiency and was used in the following experiments. The negative control (shCTL) used was a shRNA inhibiting luciferase gene expression.

**c** Western blot analysis protein level of caspase-8(P43, P18), cleaved-PARP, caspase-3 and cleaved-caspase-3 in LNCaP caspase-8-HA transient overexpression cells. Cells were treated with 20 μM Z-vad FMK for 72 h.

**d** Morphology of LNCaP transiently overexpressed caspase-8 in bright field (Mentioned in Fig S2-c). Scale bar: 50 μm.

**e** Cells were treated with 10 μM enzalutamide or DMSO vehicle for 8 days, Western blot analysis protein level of caspase-8 in LNCaP Luc-HA cells transfected with sh*CASP8* by lentivirus.

**f** Cell survival (%) of LNCaP Luc-HA (transfected with sh*CASP8* by lentivirus) cells were analyzed under enzalutamide-treatment. 5×10^4^ of LNCaP luc-HA cells were seeded in 6-well plates. 12 h later, cells were infected with sh*CASP8* virus or shCTL virus, and treated with 10 μM enzalutamide or DMSO vehicle for 8 days under 2% FBS in culture media. Values were normalized to DMSO-treated control cells (n = 3).

**g** Western blot analysis the efficacy of knocking down caspase-8 in VCaP WT and VCaP ENZR cells transfected with sh*CASP8* and shCTL by lentivirus.

**h** Cell survival (%) curve of VCaP cells treated with enzalutamide. 2×10^4^ VCaP cells (VCaP WT shCTL/sh*CASP8*, VCaP ENZR shCTL/sh*CASP8*) were seeded in 96-well plates. 24 h later, all cells were treated with or without 1 μM enzalutamide. Cell viability was analyzed using CCK-8 and normalized to the drug-free condition (n = 3).


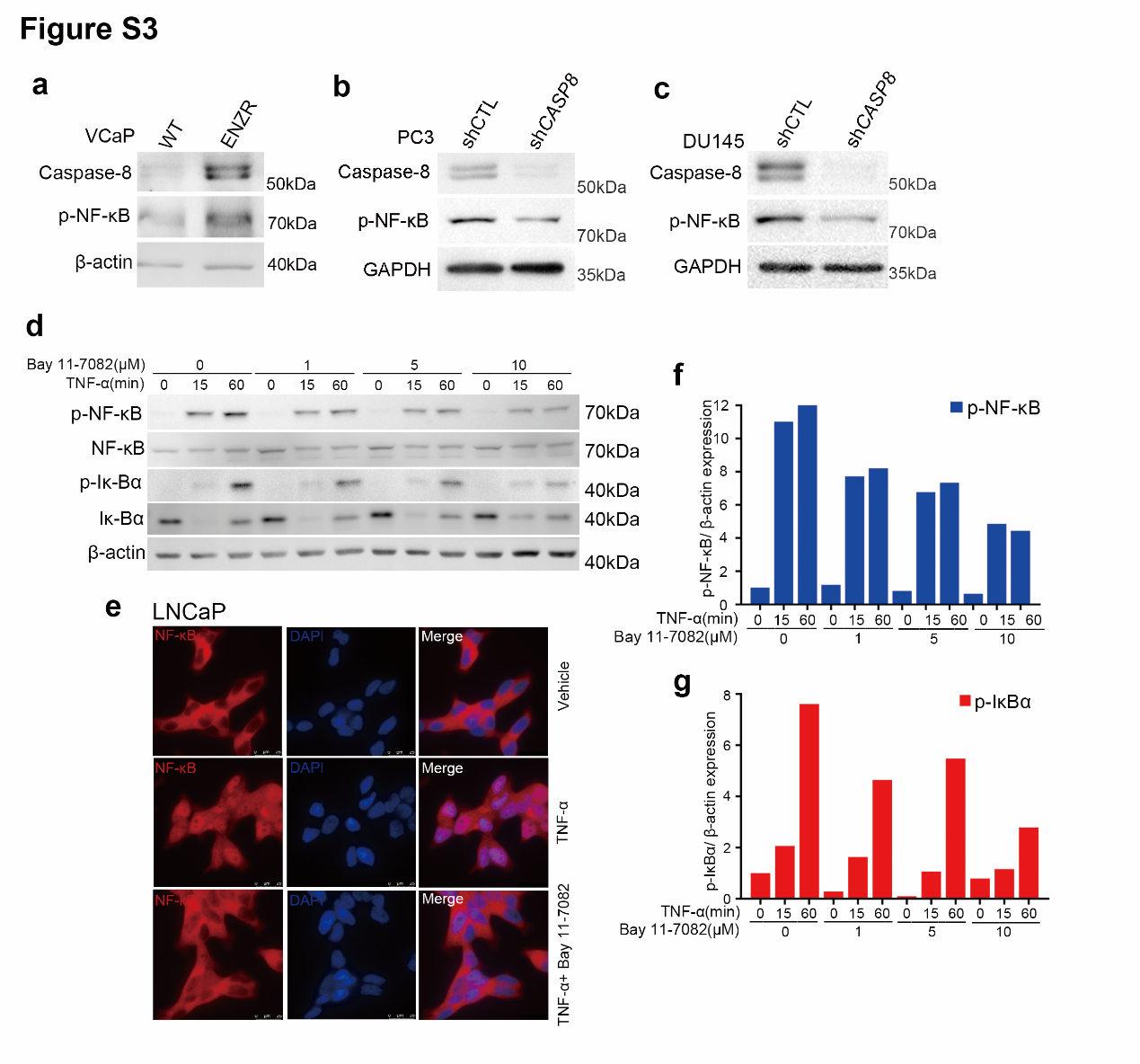


**Supplementary Fig. 3. Activation of NF-κB pathway in LNCaP cells**

**a** Western blot analysis protein level of caspase-8 and phosphorylation of NF-κB in VCaP WT and VCaP ENZR cells under serum starvation for 24 h.

**b** Western blot analysis protein level of caspase-8 and phosphorylation of NF-κB in PC3 sh*CASP8* and PC3 shCTL cells under serum starvation for 24 h.

**c** Western blot analysis protein level of caspase-8 and phosphorylation of NF-κB in DU145 sh*CASP8* and DU145 shCTL cells under serum starvation for 24 h.

**d** LNCaP WT cells were treated with 15 ng/ml hTNF-α in growth medium with 2% FBS for 15 min or 60 min. cells were pre-incubated with 1,5,10 μM Bay 11-7082 or vehicle for 30 min before hTNF-α treatment. Proteins were subjected to analysis level of phosphorylation of NF-κB and IκBα by Western blot.

**e** Distribution of NF-κB(P65) in LNCaP cells treated with TNF-α and or Bay11-7082. LNCaP cells were treated with 15 ng/ml hTNF-α or BSA vehicle for 15 min and pre-incubated with or without 10 μM Bay 11-7082 for 30 min before hTNF-α treatment. Immunofluorescence staining for NF-κB(P65) (red) and DAPI (blue) was presented.

**f** Western blot quantification of P-NF-κB protein level. The signal intensity of P-NF-κB (mentioned in Fig S3d) was quantified using Image J and normalized to the blot in first line.

**g** Western blot quantification of P-IκBα protein level. The signal intensity of P-IκBα (mentioned in Fig S3d) was quantified using Image J and normalized to the blot in first line.


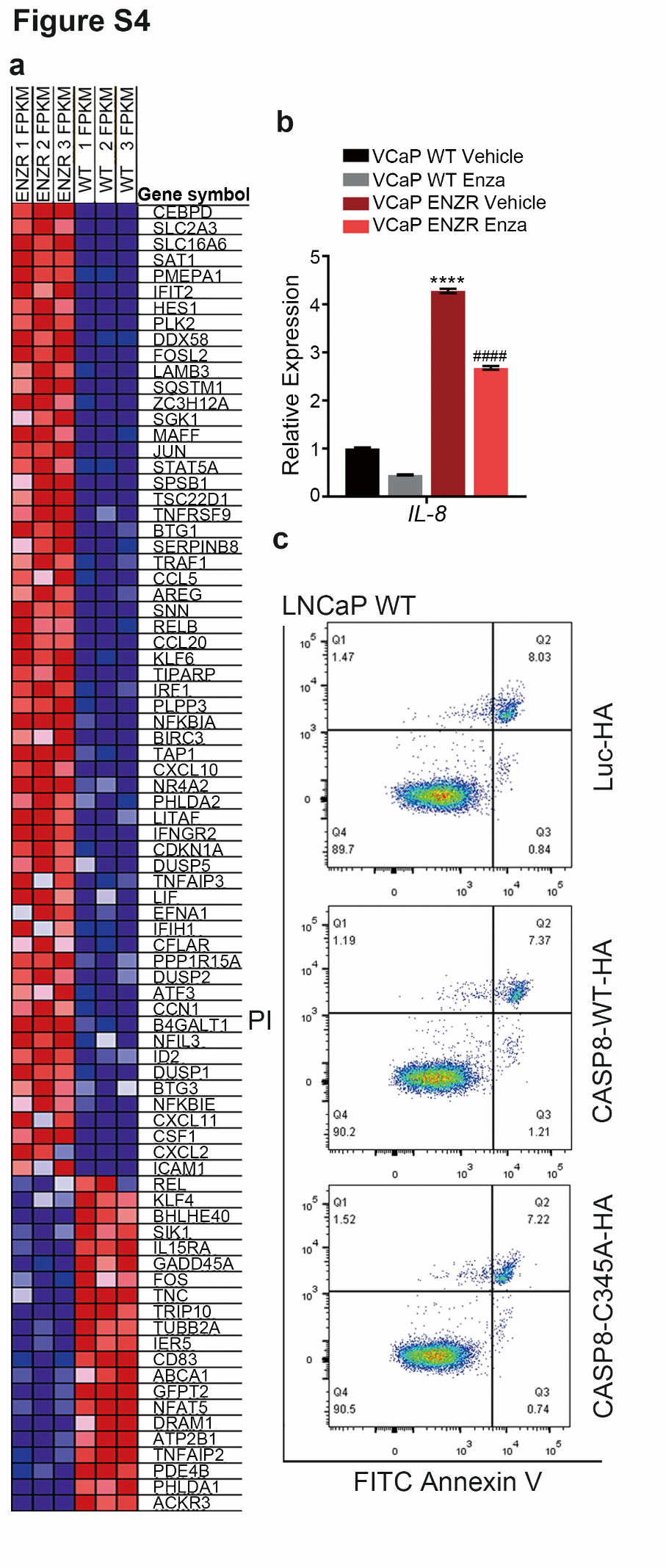


**Supplementary Fig. 4 Activation of NF-κB pathway in LNCaP cells**

**a** Heat maps of GSEA Hallmark TNF-α signaling via NF-κB profiling of LNCaP WT and ENZR cell lines. Each row represents a single gene, and each column a single cell sample. Red or green represent up-regulated or down-regulated genes (ENZR/WT) compared to the mean FPKM value on each row, respectively. Three repetitions of LNCaP WT and ENZR cell samples are displayed. Genes are ranked by rank metric score.

**b** RT-qPCR analysis of IL-8 relative expression in VCaP WT and VCaP ENZR cells under the treatment of 1 μM enzalutamide or DMSO vehicle for 48 h. GAPDH was used as internal reference. * vs. VCaP WT vehicle, # vs. VNCaP WT Enza (n = 3).

**c** Apoptosis detection of LNCaP overexpressing caspase-8-WT-HA and caspase-8-C345A-HA. LNCaP cells stably overexpressing caspase-8-WT-HA or caspase-8-C345A-HA were staining with FITC Annexin V and PI, and analyzed using flow cytometry. LNCaP luc-HA cells were used as a control.


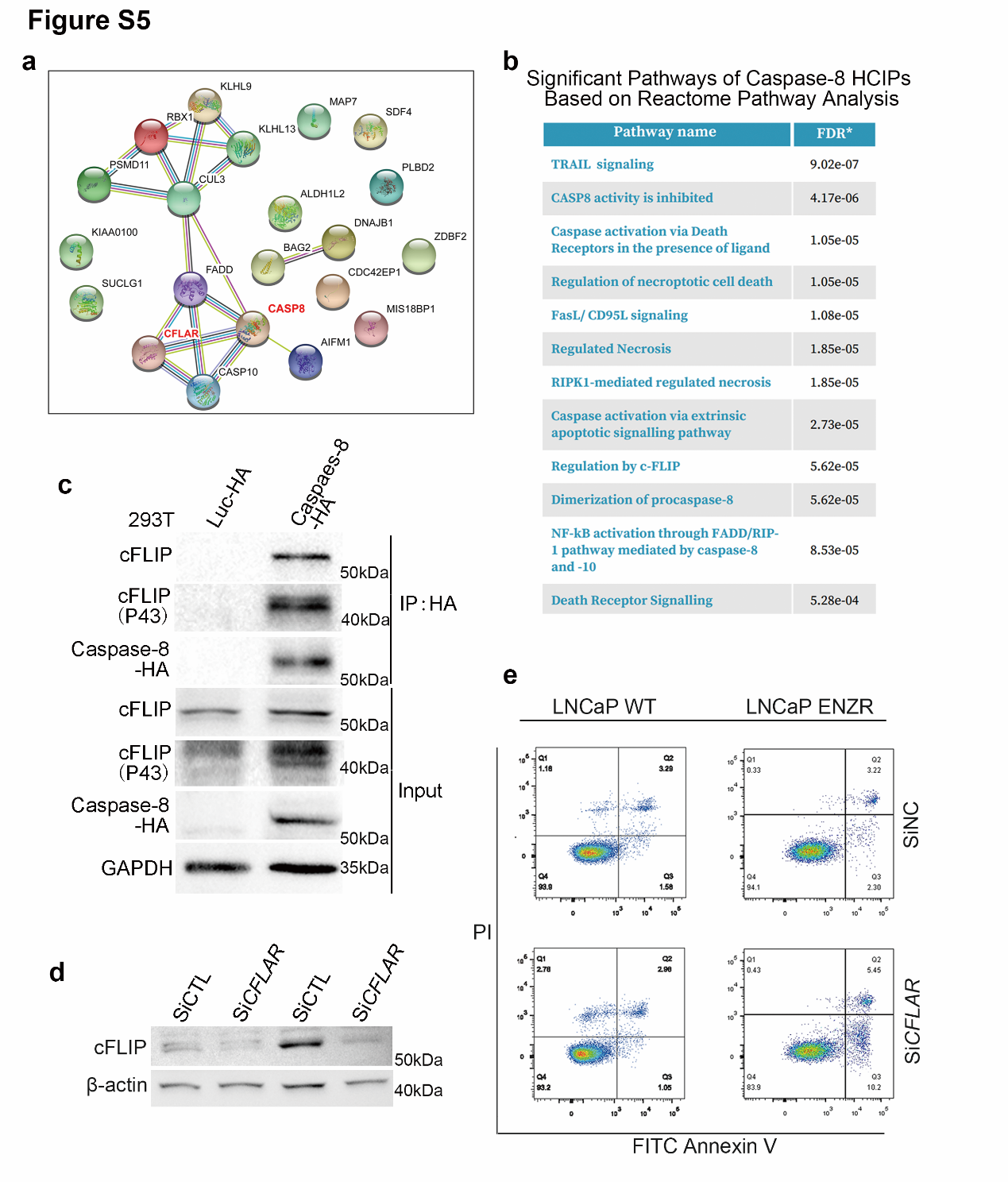


**Supplementary Fig. 5**

**The interaction of caspase-8 with cFLIP in caspase-8-mediated NF-κB activation**

**a** STRING analysis of HCIPs with caspase-8-HA in LNCaP mentioned in Figure 5a.

**b** Reactome analysis of the HCIPs with caspase-8-HA.

**c** 293T cells were stably expressed Luc-HA and caspase-8-HA by lentivirus. Cells were lysed in MCLB buffer, proteins were subjected to IP using HA magnetic beads, and co-precipitation endogenous cFLIP and cFLIP(p43) was detected by Western blot. 293T Luc-HA cells were used as a negative control.

**d** LNCaP ENZR and WT cells were transfected with 100 nM SiCTL and Si*CFLAR* 2^#^ for 3 days, protein were harvested for western blot to valuating the knock down efficiency of Si*CFLAR*.

**e** Apoptosis analysis of LNCaP WT ENZR and LNCaP ENZR cells transfected with 100 nM SiCTL and Si*CFLAR* for 3 days. Cells were harvested, stained with FITC Annexin V and PI and analyzed using flow cytometry. Scramble siCTL was used as a negative control.


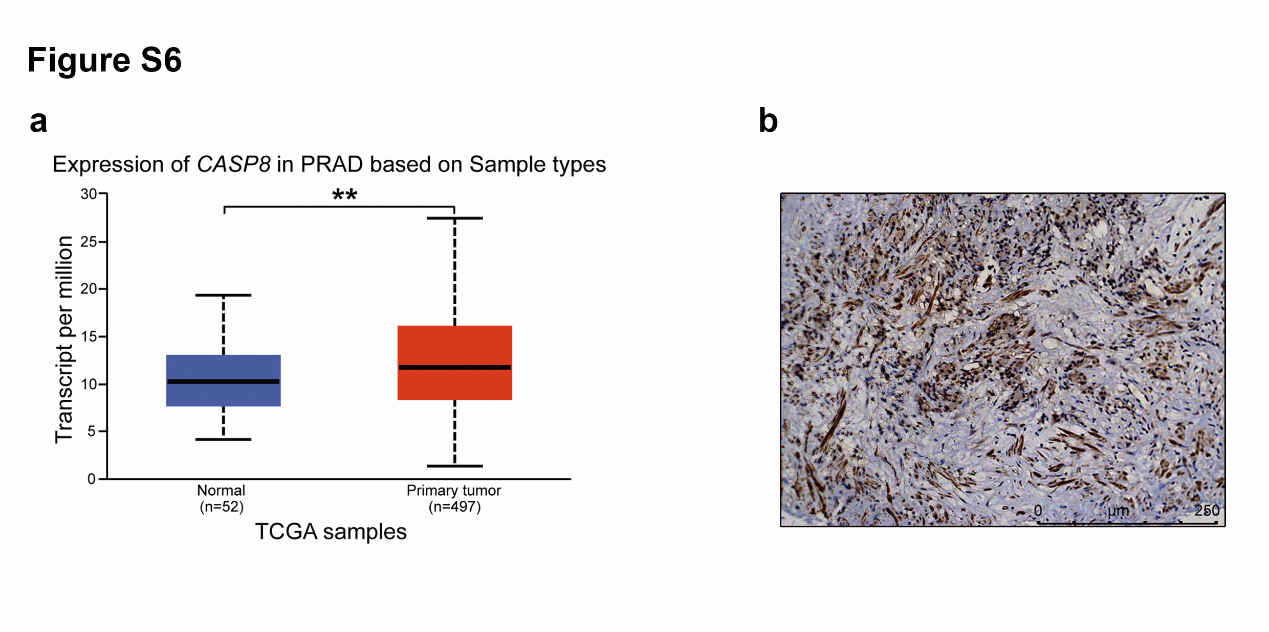


**Supplementary Fig. 6**

**Caspase-8 levels correlate positively with prostate cancer clinical progression**

**a** Caspase-8 transcriptional levels in prostate tissue and primary prostate adenocarcinoma (PRAD). Caspase-8 was significantly upregulated in primary prostate adenocarcinoma compared to normal prostate tissue in TCGA expression data.

**b** Representative images of immunohistochemical staining for cytosolic and nuclear caspase-8 positive in PCa (GS: 5+4), Scale bar: 250 μm.

**Supplementary Table 1. Genes’ FPKM in RNA-seq**

| **Gene name** | **LNCaP WT** | **LNCaP ENZR** | **log2FC** | **q-value** |
| --- | --- | --- | --- | --- |
| *CASP8* | 1.6256 ± 0.0774 | 2.9220 ± 0.1419 | 0.85 | < 0.0001 |
| *CFLAR* | 0.5167 ± 0.0302 | 1.1282 ± 0.2193 | 1.13 | < 0.0001 |
| *IL8* | 0.0000 ± 0.0000 | 0.4228 ± 0.0381 | / | < 0.0001 |
| *CXCR1* | 0.0000 ± 0.0000 | 0.1000 ± 0.0277 | / | < 0.01 |
| *CXCR2* | 0.0064 ± 0.0064 | 0.0667 ± 0.0216 | 3.38 | < 0.05 |

CASP8, CFLAR, IL-8, CXCR1 and CXCR2 FPKM are presented as Mean ± SEM. Log_2_FC = Log_2_[Mean of FPKM (LNCaP ENZR/LNCaP WT)]. Software edgeR was used to analyze statistical differences (q-value) comparing LNCaP WT and LNCaP ENZR cells, based on RNA seq data.

**Supplementary Table 2.**

**Association of Caspase-8 staining score and frequency with** **clinicopathological parameters of prostate cancer patients.**

| **Parameter** | **Variable** | **n=** | **Cytoplasmic** | | **P value** |
| --- | --- | --- | --- | --- | --- |
|  |  |  | **Low** | **High** |  |
| Gleason Score | ≤4+3 | 33 | 17 | 16 | 0.2362 |
|  | ≥4+4 | 30 | 11 | 19 |  |
| Clinical TNM Category | I/II | 19 | 13 | 6 | 0.1769 |
|  | III/IV | 44 | 22 | 22 |  |
| Positive Nuclear | Positive | 11 | 2 | 9 | 0.0537 |
|  | Negative | 52 | 26 | 26 |  |
| Pre-operative PSA | ＜4 | 4 | 3 | 1 | 0.2840 |
|  | 4-20 | 18 | 12 | 6 |  |
|  | ≥20 | 36 | 17 | 19 |  |

**Supplementary Table 3. Antibody list**

| **Antibodies** | **SOURCE** | **IDENTIFIER** | **Application/Dilutions** |
| --- | --- | --- | --- |
| Mouse-Caspase-8 | CST | #9746 | IB 1:500 |
| Rabbit-Caspase-3 | CST | #9662 | IB 1:500 |
| Rabbit-Cleaved Caspase-3 (Asp175) | CST | #9661 | IB 1:1000 |
| Mouse-IκBα | CST | #4814 | IB 1:1000 |
| Rabbit-Phospho-IKKa/b (Ser176/180) | CST | #2697 | IB 1:1000 |
| Rabbit-Phospho-IκBα (Ser32) | CST | #2859 | IB 1:1000 |
| Rabbit-Phospho-NF-κB p65(Ser536) | CST | #3033 | IB 1:1000 |
| Rabbit-NF-κB | CST | #8242 | IB 1:1000; IF 1:500 |
| Rabbit-AR | Abcam | ab74272 | IB 1:1000 |
| Rabbit-AR | CST | #5153 | IB 1:1000 |
| Rabbit-Cleaved PARP (Asp214) | CST | #5625 | IB 1:1000 |
| Mouse [-β-Actin](http://www.baidu.com/link?url=2SrvDTe-MWZb9mX3ILCjsUPiCthjkBpnSFRgNnW73U_R-bQnbPke7_nalJAWdQ56XxuZAmPY-yeTE_y4xB73BOiotkbFGSbNJj6S2icgCm7) | ProteinTech | [66009-1](http://www.baidu.com/link?url=2SrvDTe-MWZb9mX3ILCjsUPiCthjkBpnSFRgNnW73U_R-bQnbPke7_nalJAWdQ56XxuZAmPY-yeTE_y4xB73BOiotkbFGSbNJj6S2icgCm7) | IB 1:5000 |
| Mouse-GAPDH | ProteinTech | 60004-1 | IB 1:5000 |
| Rabbit-cFLIP | ProteinTech | 10394-1 | IB 1:1000 |
| Rabbit-Caspase-8 | Abcam | ab108333 | IHC 1:500 |
| HA-tag [HRP], pAb, Goat | Genscript | A00169 | IB 1:2000 |
| Mouse-V5-tag | ProteinTech | 14440-1 | IB 1:5000 |

**Supplementary Table 4. ShRNA/SiRNA list**

| SiRNA/ShRNA | Sequence (5’-3’) |
| --- | --- |
| Sh*CASP8*-1^#^ | 5’-gcacagtagagcaaatctaTTCAAGAGAtagatttgctctactgtgc-3’ |
| Sh*CASP8*-2^#^ | 5’-cctggtacatccagtcactTTCAAGAGAagtgactggatgtaccagg-3’ |
| Sh*CASP8*-3^#^ | 5’-gtgcccaaacttcacagcaTTCAAGAGAtgctgtgaagtttgggcac-3’ |
| si*CFLAR* 1^#^ | 5'-gauaagcaaggagaagaguuu-3' |
| si*CFLAR* 2^#^ | 5'-gcagucuguucaaggagcauu-3' |

**Supplementary Table 5. qPCR primer list**

| **qPCR Primer** | **Sequence (5’-3’)** |
| --- | --- |
| CASP8-F | TCATGGACCACAGTAACATGGA |
| CASP8-R | AGTGAACTGAGATGTCAGCTCAT |
| AR-F | TCCATCTTGTCGTCTTCGGAA |
| AR-R | GGGCTGGTTGTTGTCGTGT |
| AR-V7-F | CCATCTTGTCGTCTTCGGAAATGTTATGAAGC |
| AR-V7-R | TTTGAATGAGGCAAGTCAGCCTTTCT |
| GAPDH-F | CTGGCCAAGGTCATCCATGAC |
| GAPDH-R | CTTGCCCACAGCCTTGGCAG |
| TMPRSS2-F | GTCCCCACTGTCTACGAGGT |
| TMPRSS2-R | CAGACGACGGGGTTGGAAG |
| PSA-F | GACCAAGTTCATGCTGTGTGC |
| PSA-R | CCACTCACCTTTCCCCTCAAG |
| NKX3.1-F | CCCACACTCAGGTGATCGAG |
| NKX3.1-R | GAGCTGCTTTCGCTTAGTCTT |
| IL-8-F | TTTTGCCAAGGAGTGCTAAAGA |
| IL-8-R | AACCCTCTGCACCCAGTTTTC |
| CFLAR-F | TCAAGGAGCAGGGACAAGTTA |
| CFLAR-R | GTTGAGCGCCAAGCTGTTC |
| CCL5-F | CCTGCTGCTTTGCCTACATT |
| CCL5-R | GGGTGACAAAGACGACTGCT |
| CCL20-F | TGCTGTACCAAGAGTTTGCTC |
| CCL20-R | AGCATTGATGTCACAGCCTTC |
| CXCL10-F | GTGGCATTCAAGGAGTACCTC |
| CXCL10-R | TGATGGCCTTCGATTCTGGATT |
